# Non-coding RNA derived from the region adjacent to the human *HO-1* E2 enhancer selectively regulates *HO-1* gene induction by modulating Pol II binding

**DOI:** 10.1093/nar/gku1169

**Published:** 2014-11-17

**Authors:** Atsushi Maruyama, Junsei Mimura, Ken Itoh

**Affiliations:** Department of Stress Response Science, Hirosaki University Graduate School of Medicine, 5 Zaifu-cho, Hirosaki 036-8562, Japan

## Abstract

Recent studies have disclosed the function of enhancer RNAs (eRNAs), which are long non-coding RNAs transcribed from gene enhancer regions, in transcriptional regulation. However, it remains unclear whether eRNAs are involved in the regulation of human heme oxygenase-1 gene (*HO-1*) induction. Here, we report that multiple nuclear-enriched eRNAs are transcribed from the regions adjacent to two human *HO-1* enhancers (i.e. the distal E2 and proximal E1 enhancers), and some of these eRNAs are induced by the oxidative stress-causing reagent diethyl maleate (DEM). We demonstrated that the expression of one forward direction (5′ to 3′) eRNA transcribed from the human *HO-1* E2 enhancer region (named *human HO-1enhancer RNA E2-3*; hereafter called *eRNA E2-3*) was induced by DEM in an NRF2-dependent manner in HeLa cells. Conversely, knockdown of BACH1, a repressor of *HO-1* transcription, further increased DEM-inducible *eRNA E2-3* transcription as well as *HO-1* expression. In addition, we showed that knockdown of *eRNA E2-3* selectively down-regulated DEM-induced *HO-1* expression. Furthermore, *eRNA E2*-3 knockdown attenuated DEM-induced Pol II binding to the promoter and E2 enhancer regions of *HO-1* without affecting NRF2 recruitment to the E2 enhancer. These findings indicate that *eRNAE2-3* is functional and is required for *HO-1* induction.

## INTRODUCTION

Heme, which is conserved from prokaryotes to eukaryotes, is a biological cofactor of hemoproteins, including hemoglobin and cytochrome c, which are involved in oxygen transport and electron transfer reactions, respectively (1). Because intracellular free heme is highly toxic to cells due to the Fenton reaction, excess heme (e.g. derived from intravascular hemolysis or increased in several diseases) causes cell damage, while the lack of it retards the biological function of hemoproteins (1). Thus, the synthesis and degradation of heme are strictly controlled in cells (2).

Heme oxygenase (HO) catalyzes the oxidative degradation of heme into biliverdin, ferrous iron and carbon monoxide (CO) (3). Biliverdin is then readily reduced to bilirubin by biliverdin reductase that acts as an antioxidant. CO functions as a second messenger that controls the inflammatory response and apoptosis (4). Two isoforms of HO have been characterized in human cells: inducible HO-1 and constitutive HO-2 (5,6). HO-1 has multiple biological roles, such as heme detoxification, iron recycling, oxidative stress response and regulation of inflammation (1,7,8). Therefore, HO-1 is thought to be a potential therapeutic target for many diseases.

*HO-1* gene (*HO-1*) expression responds very sensitively to its substrate heme (9), as well as to multiple environmental stressors, including oxidative stress (10), heavy metals (11) and nitric oxide (12). *HO-1* induction is dependent upon gene enhancers located approximately 4 and 9 kb upstream of the transcriptional start site in human cells; these are called enhancer 1 (E1) and enhancer 2 (E2), respectively (13,14). These enhancer regions contain several binding sites for transcription factors, including multiple stress response elements (StREs) that overlap with the TPA-responsive element (TRE) and antioxidant-responsive elements (AREs) (15). *HO-1* expression is regulated by the balance between the activator NRF2 (nuclear factor erythroid-derived 2-related factor 2) and the repressor BACH1 (BTB and CNC homology 1), both of which bind to StRE. In the resting state, the BACH1/small Maf proteins (Mafs) heterodimer interacts with *HO-1* enhancer regions and represses *HO-1* expression (16). In response to environmental stimuli, BACH1 is released from *HO-1* enhancer regions, and the NRF2/small Mafs heterodimer binds to the AREs in E1 and E2 enhancers to up-regulate expression of *HO-1* (17).

NRF2 is an environmental stress-responsive transcriptional activator that is conserved from nematodes to mammals (18,19). NRF2 activates the expression of more than a hundred genes, including *HO-1* and thioredoxin reductase 1 (*TXNRD1*) (20). Most likely reflecting the multiplicity of the inducers, enhancer-dependency and the high-stress sensitivity of *HO-1* induction, several specific factors are involved in NRF2-mediated *HO-1* induction. We previously reported that BRG1 (Brahma-related gene 1), a core subunit of the chromatin remodeling complex, and the actin motif of the Neh5 domain of NRF2 are selectively involved in the induction of *HO-1* (21,22). We demonstrated that BRG1 binds to the *HO-1* enhancer and activates *HO-1* transcription by enhancing Z-DNA formation in its promoter region (21). Using a molecular probe that specifically detects Z-DNA, we recently showed that Z-DNA formation precedes *HO-1* induction in cultured cells (23). However, the molecular mechanisms of enhancer- and NRF2-mediated *HO-1* induction are not fully understood.

In 2005, Carninci *et al.* described the comprehensive identification of transcription start and termination sites as well as previously unidentified, full-length cDNAs derived from the mouse and human genomes (24). Moreover, recent large-scale genome-wide sequencing studies, such as the ENCODE project, revealed that three-quarters of the human genome is transcribed (25). Importantly, these studies show that non-coding regions, including enhancer, promoter and intergenic regions, are transcribed and generate non-coding RNAs (ncRNAs). Although most characterized ncRNAs are ribosomal and transfer RNAs, ncRNAs are classified into subclasses, such as long ncRNA (lncRNA), microRNA (miRNA) and small nucleolar RNA, based on their length, origin and localization (26,27). These ncRNAs participate in the regulation of transcription, translation and diseases (28). It has been reported that miRNAs, including miR-155, miR-377, miR-217 and miR-122, regulate HO-1 protein expression in cultured human cells (29–31). However, it is unknown whether lncRNAs are involved in the regulation of human *HO-1* expression.

In this study, we focused on and analyzed the non-coding transcripts derived from enhancer regions of human *HO-1*. As a result, we demonstrated that the regions adjacent to human *HO-1* enhancers are actively transcribed and that the eRNAs are required for *HO-1* induction.

## MATERIALS AND METHODS

### Sequences of 5' Rapid Amplification of cDNA Ends (5' RACE) clones

The sequence data for the 5′ RACE clones have been deposited into the DNA Data Bank of Japan (DDBJ). Sequence names and accession numbers are as follows: *hHO-1 eRNA E2*: AB905426, *hHO-1 eRNA E2_L*: AB905427, *hHO-1 eRNA E1_1*: AB905428 and *hHO-1 eRNA E1_2*: AB905429.

### Oligonucleotide primers and human BACH1 expression plasmid

The oligonucleotide primer sequences used in this study are listed in Supplementary Table S1. The human BACH1 expression plasmid was provided by Dr. Tsutomu Toki, Department of Pediatrics, Hirosaki University Graduate School of Medicine.

### Cell culture

Human cervical carcinoma HeLa cells were cultured in RPMI 1640 medium (Sigma-Aldrich) containing 10% fetal bovine serum and 100 units/ml penicillin-streptomycin. The cells were cultured at 37°C with 5% CO_2_ and saturated humidity.

### Antibodies

Anti-NRF2 (sc-13032), anti-Lamin B (sc-6217), anti-Pol II (sc-899), anti-HSP90 (sc-59577) and anti-BACH1 (sc-14700) antibodies were purchased from Santa Cruz Biotechnology, Inc. Anti-β-Actin antibody (A1978) was obtained from Sigma-Aldrich. Anti-Pol II antibody (clone CTD4H8) and normal mouse immunoglobulin G (IgG) were purchased from Millipore. Anti-HO-1 (ab68477) was purchased from Abcam.

### Preparation of cytoplasmic and nuclear fractions

Cells were suspended in hypotonic buffer (10 mM HEPES-NaOH pH7.9, 10 mM KCl, 1.5 mM MgCl_2_, 0.5 mM DTT, 0.1% TritonX-100) containing 1× Complete (Roche) and 10 μM MG132, mixed well by pipetting and incubated on ice for 10 min. Then, cell suspensions were centrifuged at 3,000 revolutions per minute for 1 min at 4°C, and the supernatants were reserved as the cytoplasmic fraction. Nuclear pellets were washed additional twice more with hypotonic buffer and the final pellets were reserved as the nuclear fraction. For immunoblot analysis, samples were denatured by boiling with 2-mercaptoethanol and sodium dodecyl sulphate (SDS) and were then subjected to SDS-polyacrylamide gel electrophoresis (SDS-PAGE). For reverse transcriptase-polymerase chain reaction (RT-PCR) analysis, RNA samples were isolated by using TRIzol reagent LS (Life Technologies) and Nucleospin RNA II (TaKaRa Bio), and cDNAs were synthesized as described below.

### Real-time RT-PCR analysis

Total RNA was isolated from cultured cells using Nucleospin RNA II (TaKaRa Bio) according to the manufacturer's protocol. The cDNAs were synthesized using PrimeScript^®^ RTase (TaKaRa Bio) using total RNA as a template and random hexamers. The real-time RT-PCR analysis was performed using Premix Ex Taq™ II (Perfect Real Time) (Takara Bio) with a CFX Real-Time PCR Detection System (Bio-Rad). The expression of *HO-1*, *TXNRD1* and glutamate-cysteine ligase catalytic subunit (*GCLC*) was measured by real-time RT-PCR using the TaqMan^®^ Gene Expression Assay (Applied Biosystems). The cyclophilin A gene (*CycA*) was used as an internal control and was analyzed using the TaqMan^®^ Gene Expression Assay (Applied Biosystems). The RNA levels of the*hHO-1 eRNAs* were measured by real-time RT-PCR according to the Universal Probe Library system (Roche) with the following primers and Universal Probe Library Probes (Roche): *hHO-1 eRNA E2-1* (*eRNA E2-1*): 5′- AAA AAG TTC CCA CGG TGC T -3′, 5′- CAC CGG GTC CCT TAA CAA A -3′ and Probe #53; *hHO-1 eRNA E2-2* (*eRNA E2-2*): 5′- GCT CAC TTC TGG GCT CAC TTA -3′, 5′- GAC TCT CTA AGC TCT AAA GGG TGG T -3′ and Probe #66; *hHO-1 eRNA E2-3* (*eRNA E2-3*): 5′- GTC TGG GGC CTG AAT CCT A -3′, 5′- GGC TAG AGG AGG AGT GAG AGG -3′ and Probe #17; *hHO-1 eRNA E1-4* (*eRNA E1-4*): 5′-TGA AAG GGC AGC TTT AAT GG -3′, 5′- GAG GCT TCT GCC GTT TTC TA -3′ and Probe #51. To normalize the *hHO-1 eRNA*s levels, the cyclophilin A gene was used as an internal control and measured using the PPIA Universal probe library set (Roche). The gene expression of solute carrier family 7 member 11 (*SLC7A11*) (anionic amino acid transporter light chain, x_c_^−^ system), ferritin light polypeptide (*FTL*) and sequestosome 1 (*SQSTM1*) was measured by SYBR Green real-time RT-PCR using SYBR^®^ Premix Ex Taq™ II (Tli RNaseH Plus) (Takara Bio) and the primers listed in Supplementary Table S1.

### Chromatin immunoprecipitation (ChIP) assay

ChIP assays were performed as described (32). Briefly, cells were fixed with 1% formaldehyde for 10 min at room temperature. Subsequently, glycine was added to 0.125 M (final concentration), and the cells were incubated for 5 min at room temperature. The fixed cells were dissolved in cell lysis buffer, sonicated and centrifuged, and the supernatant fraction was used as the cell lysate for ChIP. Antibodies against each target protein were immobilized to DynaBeads Protein G (Life Technologies) and used for immunoprecipitation. Precipitated DNA fragments were detected by SYBR Green real-time PCR with the primer sets listed in Supplementary Table S1.

### siRNA transfection

Stealth siRNAs against *eRNA E2-3*, *NRF2* and *BACH1* were synthesized by Invitrogen with the following sequences: *eRNA E2-3* (1): 5′- CAA CCU AAA GGU GGG AGC UAC UCA A-3′, *eRNA E2-3* (2): 5′- UGC UAU GGU UUC CCU AGG AUU CAG G-3′. *NRF2* (1): 5′- CCA ACC AGU UGA CAG UGA ACU CAU U-3′, *NRF2* (2): 5′- CAA ACU GAC AGA AGU UGA CAA UUA U-3′. *BACH1* (1): 5′-GGG CAC CAG GGA AGA UAG UAG UGU U-3′, *BACH1* (2): 5′- GGU CAA AGG ACU UUC ACA ACA UUA A-3′. A stealth control siRNA was obtained from Invitrogen. Then, siRNA (final 20 nM) was transfected to cultured cells using the Lipofectamine RNAi MAX reagent according to the manufacturer's protocol (Life Technologies). Twenty-four hours after transfection, the cells were subcultured and exposed to 100 μM diethyl maleate (DEM) for the appropriate time.

### Statistical analysis

The results are expressed as the mean ± SEM, and statistical significance was determined by one-way ANOVA followed by a Dunnett's *post hoc* test for multiple parameter comparisons to the control or Student's *t-*test for two parameter comparisons. A *P* < 0.05 was considered statistically significant.

## RESULTS

### RNA polymerase II (Pol II) binds to the promoter and enhancer regions of human *HO-1*

**Figure 1.**
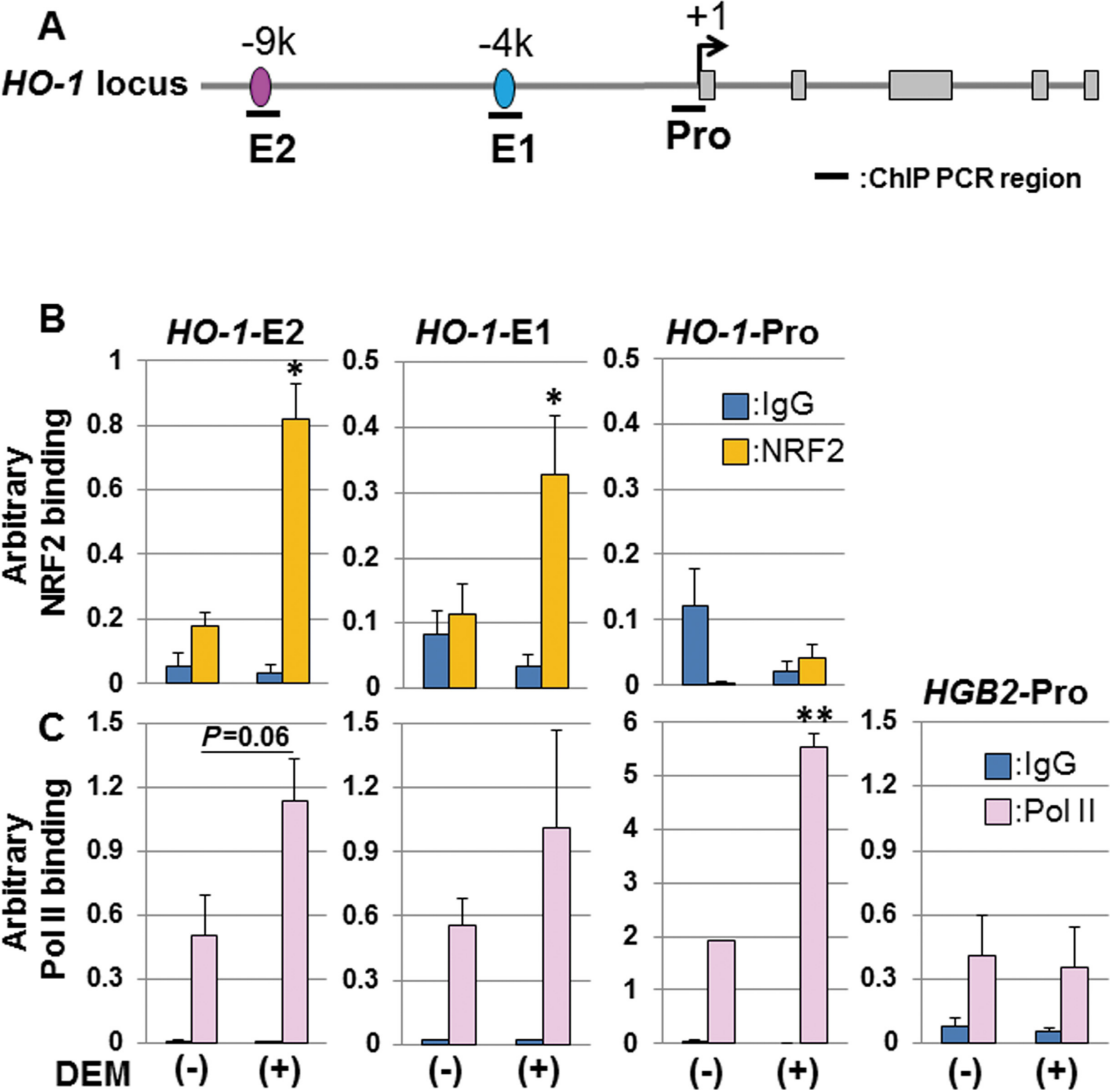
RNA polymerase II (Pol II) binds to the promoter and enhancer regions of the *HO-1* locus. (A) A schematic figure of the human *HO-1* locus. +1: transcription start site; E2: E2 enhancer region; E1: E1 enhancer region; Pro: promoter region. ChIP PCR regions are indicated by bold lines. *HO-1* exons are shown in gray boxes. The distance in kilobases (K) from the transcription start site is shown on the enhancer regions. (B) NRF2 binding to *HO-1* enhancer regions is induced by DEM administration. HeLa cells were either untreated (-) or exposed to 100 μM DEM for 3 h (+) and cell lysates were prepared. ChIP assays were performed using an anti-NRF2 antibody (yellow bars). Normal mouse IgG was used as a negative control (blue bars). (C) RNA Pol II binds to the promoter and enhancer regions of the *HO-1* locus. ChIP assays were performed using an anti-Pol II antibody (Millipore, clone CTD4H8) (pink bars). Normal mouse IgG was used as a negative control (blue bars). Binding of NRF2 or Pol II was measured by real-time PCR using specific primers for the regions shown in A. *HO-1*-E2: *HO-1* E2 enhancer region; *HO-1*-E1: *HO-1* E1 enhancer region; *HO-1*-Pro: *HO-1* promoter region; *HGB2*-Pro: γ-globin promoter region. Arbitrary binding values are expressed as the mean ± SEM of four independent assays. *P < 0.05; **P < 0.01 (two-tailed unpaired Student's *t*-test).

The inducible binding of Pol II to the human *HO-1* promoter region is a critical event for *HO-1* induction (23,33). Thus, we investigated the binding dynamics of Pol II and NRF2 to the *HO-1* gene locus in response to the NRF2 activator DEM using ChIP assays of HeLa cells. As reported previously, we detected inducible binding of NRF2 to the E2 and E1 enhancers of *HO-1*, but not to the *HO-1* promoter region, in response to DEM (Figure 1A and B). We observed Pol II binding to the enhancer regions even in the absence of DEM. We also detected a significant induction of Pol II binding to the *HO-1* promoter (Figure 1A and C) (33). Interestingly, we found that there is an increase in the ChIP signal for Pol II at the *HO-1* enhancers in the presence of DEM that is not statistically significant but in the case of E2 seems to be reproducible (Figure 1A and C). As a control, Pol II binding to the γ-globin (*HGB2*) promoter region was lower than to both *HO-1* enhancers and was not affected by DEM (Figure 1C). According to the above observation, we hypothesized that the enhancer regions of human *HO-1* are transcribed by Pol II.

### Identification of ncRNAs derived from human *HO-1* enhancer regions

To evaluate the significance of Pol II binding to *HO-1* enhancer regions in human cells, we first investigated the ChIP data for the human *HO-1* locus in the UCSC Genome Browser database (34). These data indicated that Pol II binds to two *HO-1* enhancer regions in various human cells. Furthermore, in addition to histone H3 K4 mono-methylation, which is the mark of an enhancer, we observed the active promoter mark, histone H3 K4 tri-methylation, on both enhancer regions of the *HO-1* locus in several human cultured cells, including K562 cells.

To investigate whether the enhancer regions are transcribed, we searched for cDNA tags derived from the *HO-1* locus other than those in the protein-coding region using the comprehensive cDNA database, human Cap Associated Gene Expression (CAGE) (24). We found four tags upstream of the *HO-1* transcriptional start site in the CAGE database (Supplementary Figures S4D and S5E); two were derived from the region adjacent to the E2 enhancer, one was from the region between the E2 and E1 enhancers and the fourth was within the E1 enhancer. In addition, recent RNA-sequencing data from the UCSC Genome Browser and FANTOM 5 indicate that human *HO-1* enhancers are potential active bi-directional promoters and multiple transcripts are detected adjacent to the human *HO-1* enhancer regions (35,36). These data strengthened our contention that human *HO-1* enhancer regions are actually transcribed.

**Figure 2.**
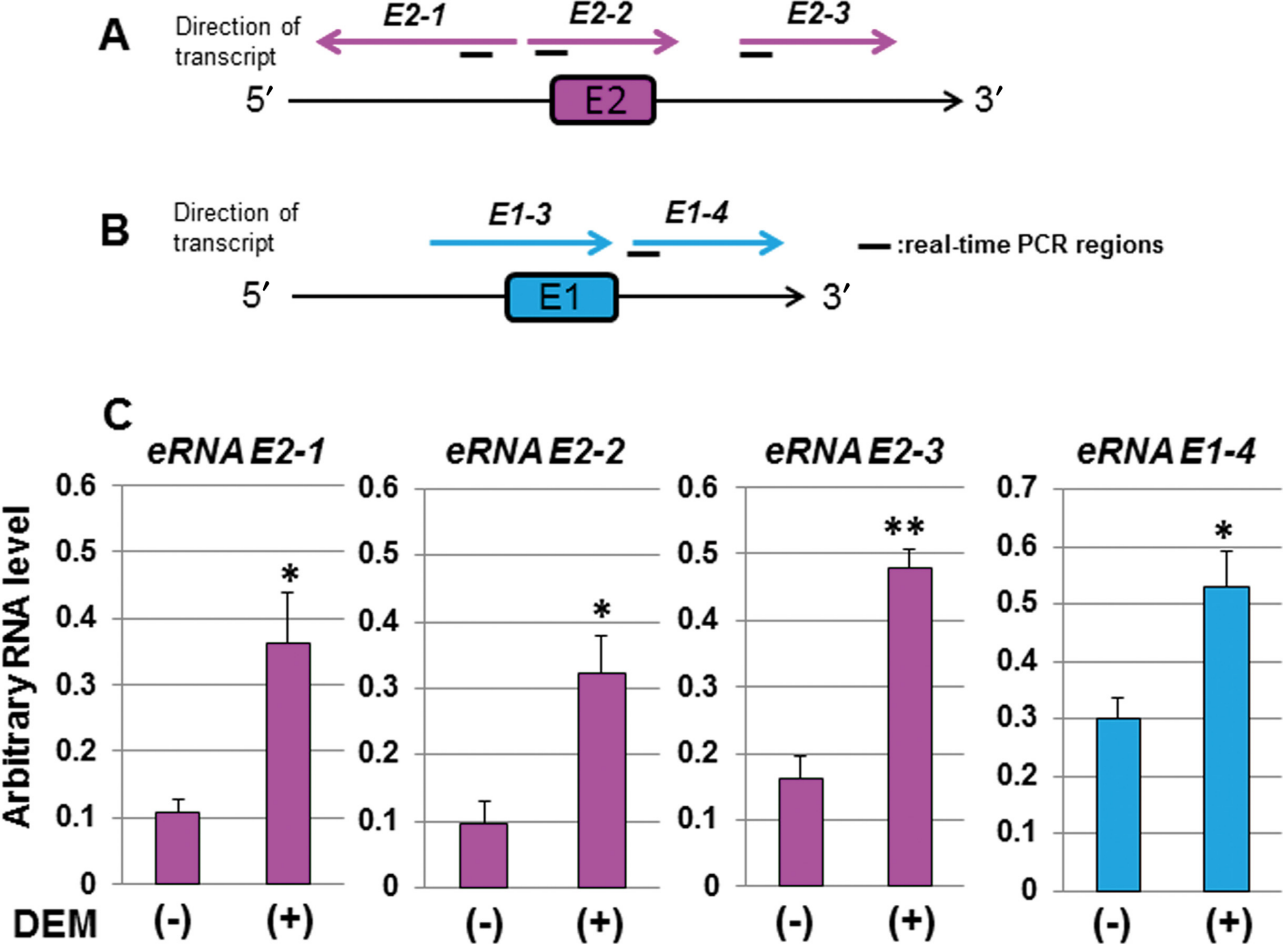
Identification of eRNAs derived from enhancer regions of the *HO-1* locus. (A and B) Transcript structure of eRNAs derived from human *HO-1* E2 enhancer (*eRNA E2-1*, *eRNA E2-2* and *eRNA E2-3*) (A) and from human *HO-1* E1 enhancer (*eRNA E1-3* and *eRNA E1-4*) (B). *eRNA E2s* and *eRNA E1s* are drawn by magenta and cyan arrows, respectively, and the direction of the RT-PCR signal is indicated by direction of the arrows. (C) The expression of *eRNA E2s* and *eRNA E1-4* in HeLa cells. HeLa cells were either untreated (−) or exposed to 100 μM DEM for 6 h (+) and total RNA samples were isolated. The cDNAs were synthesized using random hexamers with total RNA as a template. The arbitrary RNA levels were measured by real-time RT-PCR using specific primers and Universal Probe Library Probes. The value was normalized to the expression of the cyclophilin A gene, and the arbitrary RNA level was expressed as the mean ± SEM of three independent assays. *P < 0.05; **P < 0.01 (two-tailed unpaired Student's *t*-test).

To validate the presence of transcripts originating from the *HO-1* enhancer regions, we performed a transcript mapping around the human *HO-1* enhancer regions by RT-PCR analysis using total RNA from DEM-treated HeLa cells as a template and random hexamers as a cDNA synthesis primer (the detailed procedure is described in Supplementary Methods S1). As a result, we have detected three signals around E2 and two signals around E1 enhancer regions (Supplementary Figure S1). Note that PCR products were not detected in the reactions without RTase (Supplementary Figure S1). We named the RT-PCR signals derived from the region adjacent to the E2 enhancer as *human HO-1 enhancer RNA E2-1*, *human HO-1 enhancer RNA E2-2* and *human HO-1 enhancer RNA E2-3* (in this paper, we abbreviated them as *eRNA E2-1*,*eRNA E2-2* and *eRNA E2-3*, respectively). We also named the RT-PCR signals from the region adjacent to E1 as *human HO-1* e*nhancer RNA E1-3* and *human HO-1 enhancer RNA E1-4* (in this paper, we abbreviated them as *eRNA E1-3* and *eRNA E1-4*, respectively). Semi-quantitative RT-PCR results indicated that the expression of three E2-derived signals (*eRNA E2-1*, *E2-2*, and*E2-3*) and one E1-derived signal (*eRNA E1-4*) were reproducibly induced by DEM (Supplementary Figure S1B and D), and these results were confirmed by real-time RT-PCR (Figure 2C).

Next, to determine the direction of the DEM-responsive RT-PCR signals described above, we carried out strand-specific RT-PCR analysis using 5′-end-directed and 3′-end-directed internal primers for cDNA synthesis (the detailed procedure is described in Supplementary Methods S1). We found that *eRNA E2-1* is a reverse (3′ to 5′) RT-PCR signal, whereas *eRNA E2-2* and *eRNA E2-3* are forward (5′ to 3′) RT-PCR signals (Supplementary Figure S2). Similarly, we found that both *eRNA E1-3* and *E1-4* are forward RT-PCR signals (Supplementary Figure S2). Next, we examined whether there are RT-PCR signals between the E2-2 and E2-3 regions, and between the E1-3 and E1-4 regions using PCR primers overlapping the regions (Supplementary Figure S3). As a result, we detected the RT-PCR signals overlapping the E2-2 and E2-3 regions and E1-3 and E1-4 regions, respectively (Supplementary Figure S3). Therefore, we cannot conclude that *eRNA E2-2* and *eRNA E2-3* or *eRNA E1-3* and *eRNA E1-4* are independent RT-PCR signals. We summarized the detected RT-PCR products from the *HO-1* enhancer regions in Figure 2A and B. Note that we designed the forward primer for real-time RT-PCR a bit upstream of the E1-4 region that was determined by the transcript mapping analysis (Figure 2B).

To determine the transcription start site from the *HO-1* enhancer regions, we performed 5′ RACE analysis using total RNA isolated from DEM-treated HeLa cells (Supplementary Methods S1). As a result, one 5′ end of the forward-strand adjacent to the E2 enhancer and two 5′ ends of the forward-strand transcripts adjacent to the E1 enhancer were identified from multiple 5′ RACE clones (Supplementary Figures S4 and S5). The positions of the 5′ ends of the human *HO-1* eRNAs (*hHO-1 eRNAs*) and the detailed sequences are summarized in Supplementary Figures S4 and S5. The 5′-end sequence data have been deposited into the DDBJ (accession no. *hHO-1 eRNA E2*: AB905426; *hHO-1 eRNA E1_1*: AB905428; *hHO-1 eRNA E1_2*: AB905429). Moreover, to estimate the 3′ ends of the *hHO-1 eRNAs*, we conducted a 3′ primer walking analysis using HeLa cDNA and a series of primers complementary to the upstream region of human *HO-1* (Supplementary Figure S6). Finally, we identified an ∼1.3-kb transcript derived from the human *HO-1* E2 enhancer region that contains one intron; however, it is unclear at present whether this is the major full-length transcript of the E2-3 region (Supplementary Figure S7; accession no. *hHO-1 eRNA E2_L*: AB905427).

Next, to determine whether *hHO-1 eRNAs* are expressed in human cells, we investigated their expression using a real-time RT-PCR assay with total RNA samples isolated from HeLa, HaCaT and SH-SY5Y cells. We found that RT-PCR signals of *eRNA E2-1*, *eRNA E2-2* and *eRNA E2-3* are significantly induced by DEM in HeLa cells (Figure 2C). Signals from *eRNA E2-1* were also significantly induced by DEM in HaCaT and SH-SY5Y cells (Supplementary Figure S8). Signals from *eRNA E2-3* were also significantly induced by DEM in HaCaT, but not in SH-SY5Y cells (Supplementary Figure S8). The RT-PCR signal of the *eRNA E1-4* was detected in all three cells, but they were significantly up-regulated by DEM only in HeLa cells (Figure 2C and Supplementary Figure S8). In addition, the expression level of the *hHO-1 eRNAs* varied among cell types (Figure 2C and Supplementary Figure S8). These results indicate that *hHO-1 eRNAs* are actually expressed in human cells.

### DEM-induced *hHO-1 eRNAs* are enriched in the cell nucleus

**Figure 3.**
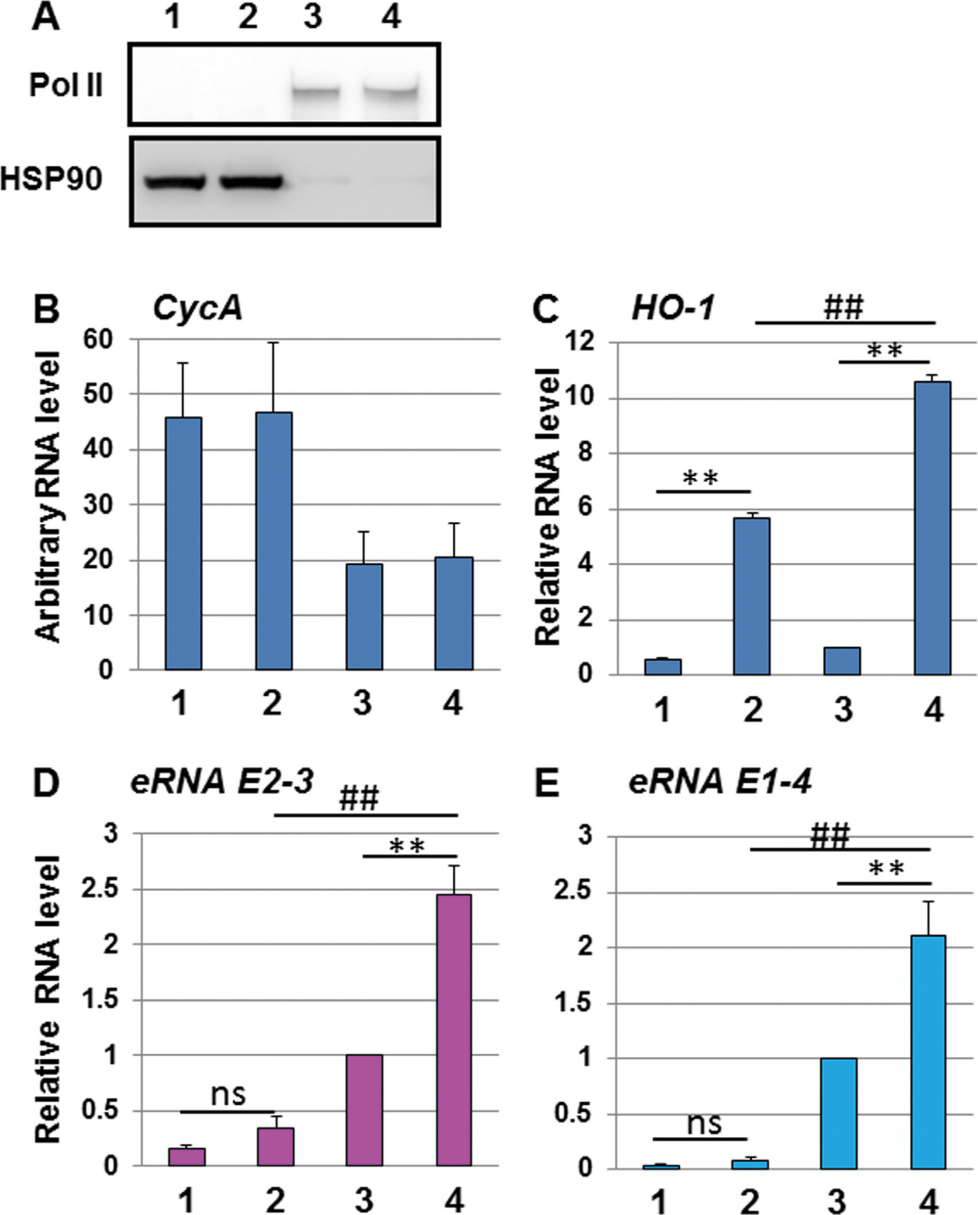
*hHO-1 eRNAs* are enriched in the cell nucleus. (A) Fractionation of the cytoplasmic and nuclear portions of HeLa cells. HeLa cells were either untreated (lanes 1 and 3) or treated with 100 μM DEM for 6 h (lanes 2 and 4) or not, and cytoplasmic (lanes 1 and 2) and nuclear portions (lanes 3 and 4) were prepared. Fractionation efficiency was validated by immunoblotting using HSP90- (Santa Cruz, sc-59577) and Pol II- (Santa Cruz, sc-899) specific antibodies as a cytoplasmic and nuclear marker, respectively. The RNA content of the cyclophilin A gene (*CycA*) (B), *HO-1* (C), *eRNA E2-3* (D) and *eRNA E1-4* (E) in the cytoplasm or the nucleus was measured by real-time PCR using either TaqMan^®^ Gene Expression Assay system or specific primer sets with Universal Probe Library Probes. The values of *HO-1*(C), *eRNA E2-3* (D) and *eRNA E1-4* (E) were normalized to *CycA* expression. The value of lane 3 was arbitrarily set as 1 and the relative RNA level was expressed as the mean ± SEM of three independent assays. **P < 0.01 (two-tailed, unpaired Student's *t*-test), ##*P* < 0.01 compared to the value of lane 2 (one-way ANOVA followed by a Dunnett's *post hoc* test for multiple parameter comparisons).

To understand the function of *hHO-1 eRNAs*, we next examined the subcellular localization of the transcripts. For this purpose, cytoplasmic and nuclear portions were fractionated from HeLa cells, and the *hHO-1 eRNAs* content was determined by real-time RT-PCR. The fractionation efficiency was verified by immunoblot analysis using anti-HSP90 and anti-Pol II antibodies as a marker of the cytoplasm and the nucleus, respectively (Figure 3A). The *CycA* RT-PCR signal, which is used as an internal control to normalize RNA levels, was approximately 2-fold higher in the cytoplasmic fraction compared to the nucleus (Figure 3B). The cytoplasmic *HO-1* signal was comparable to that of the nucleus in untreated HeLa cells, but the nuclear signal was significantly greater than the cytoplasmic signal in the DEM-treated samples (Figure 3C). On the other hand, we found that *hHO-1 eRNAs* were enriched in the nuclear fraction and significantly induced by DEM (Figure 3D and E). To examine the possibility that the detected *hHO-1 eRNAs* encode for a protein, we investigated the Kozak sequence and open reading frame (ORF) in the*hHO-1 eRNAs*. We found no Kozak sequence in the detected RT-PCR signals, and only one putative ORF that is more than 10 amino acids and contains a first methionine in the E2-3 region (Supplementary Figure S4 and S7). However, we have not found any proteins with significant similarity to the ORF. Thus, it is highly likely that *hHO-1 eRNAs* work as a ncRNA, although we cannot exclude the possibility that unannotated small peptides are expressed from *hHO-1 eRNAs*.

### The expression of *hHO-1 eRNAs* is induced by *HO-1* inducers

**Figure 4.**
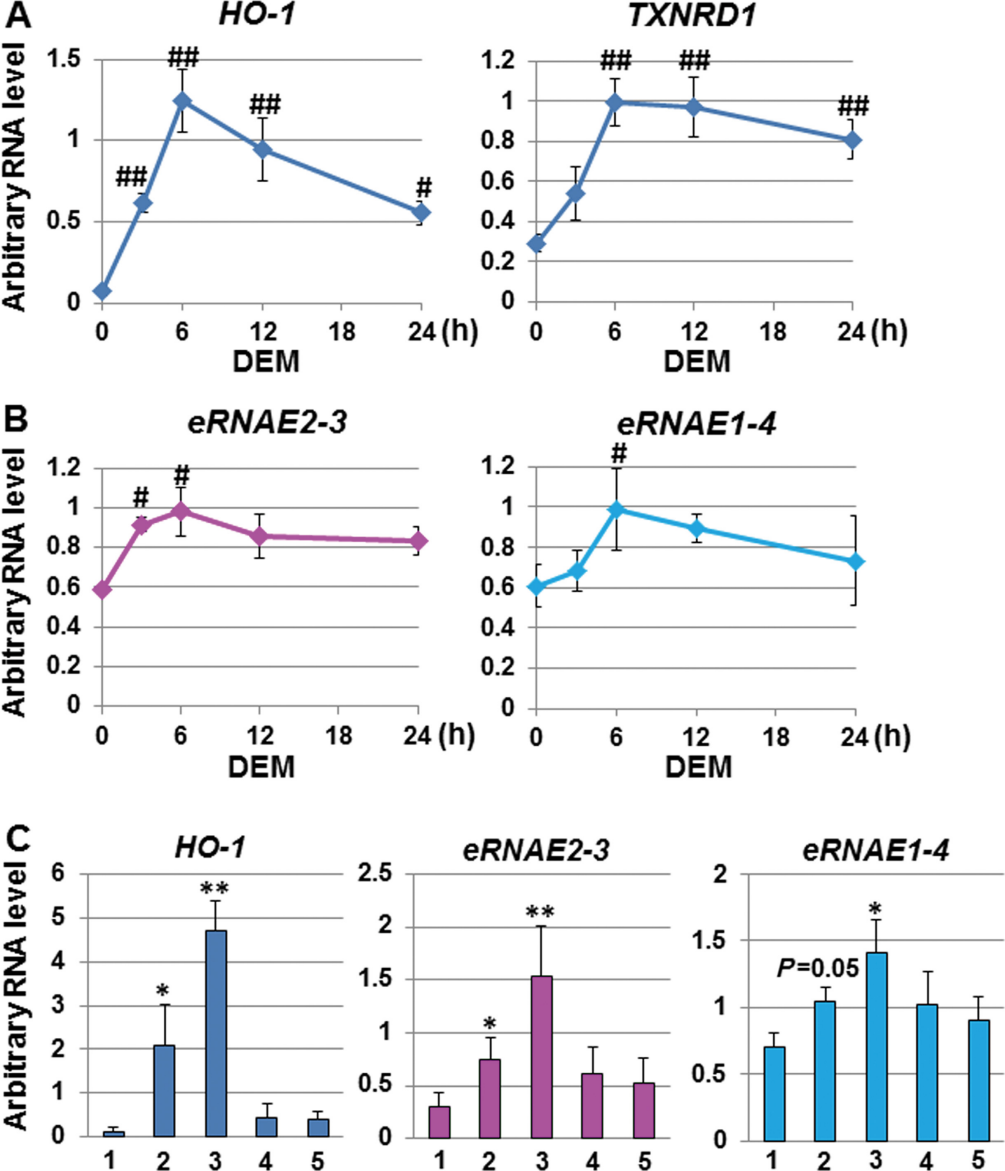
*hHO-1 eRNAs* are stress-responsive transcripts. (A) DEM-responsive expression of *HO-1* and *TXNRD1*. HeLa cells were exposed to 100 μM DEM for the indicated time (hours, h), and total RNA samples were isolated. The arbitrary RNA level of *HO-1* and *TXNRD1* was analyzed by real-time RT-PCR using the TaqMan^®^ Gene Expression Assay system. (B) DEM-responsive expression of the *hHO-1 eRNAs*. The arbitrary RNA levels of *eRNA E2-3* and *eRNA E1-4* were measured by real-time RT-PCR using specific primer sets and Universal Probe Library Probes. (C) Stress-responsive expression of *HO-1* and *hHO-1 eRNAs*. HeLa cells were exposed to various *HO-1* inducers, including control (lane 1), 100 μM DEM (lane 2), 20 μM Hemin (lane 3), 20 μM CdCl_2_ (lane 4) or 25 μM DMF (lane 5), for 6 h, and total RNA samples were isolated. The arbitrary RNA level of *HO-1*, *eRNA E2-3* and *eRNA E1-4* was analyzed by real-time RT-PCR. The values were normalized to cyclophilin A gene expression, and the arbitrary RNA level was expressed as the mean ± SEM of four independent assays. *P < 0.05; **P < 0.01 (two-tailed, unpaired Student's *t*-test), #*P* < 0.05; ##*P* < 0.01 compared to the value at 0 h (one-way ANOVA followed by a Dunnett's *post hoc* test for multiple parameter comparisons).

Because we initially found transcript tags at the near regions of *eRNA E2-3* and *eRNA E1-4* in the CAGE data, we focused on those transcripts. To investigate the time course expression profile of *hHO-1 eRNAs* in response to DEM, we performed a real-time RT-PCR analysis. We also analyzed the expression of *HO-1* and *TXNRD1* as NRF2 target genes in HeLa cells. The expression of *HO-1* and *TXNRD1* was induced by DEM and peaked after 6 h (Figure 4A). We observed that the *eRNA E2-3* and *eRNA E1-4* signals also peaked at 6 h after DEM administration. The signal of *eRNA E2-3* was significantly increased by DEM at 3 and 6 h, while that of *eRNA E1-4* was significantly increased by DEM only at 6 h (Figure 4B). However, the induction of*eRNA E1-4* by DEM was variable and the induction was not always statistically significant as shown in Figure 4C, lane 2.

We further investigated whether *hHO-1 eRNAs* were induced by various *HO-1* inducers, including Hemin, cadmium (CdCl_2_) and dimethyl fumarate (DMF), using real-time RT-PCR. The expression of *HO-1* was significantly induced by DEM and Hemin, but not by CdCl_2_ and DMF. The signal of the *eRNA E2-3* was induced by DEM and Hemin, but not by CdCl_2_ and DMF and the signal of *eRNA E1-4* was significantly induced only by Hemin. The most effective inducer of *HO-1* and *hHO-1 eRNAs* was Hemin (Figure 4C).

### Stress-inducible expression of *eRNA E2-3* is regulated by NRF2 and BACH1

**Figure 5.**
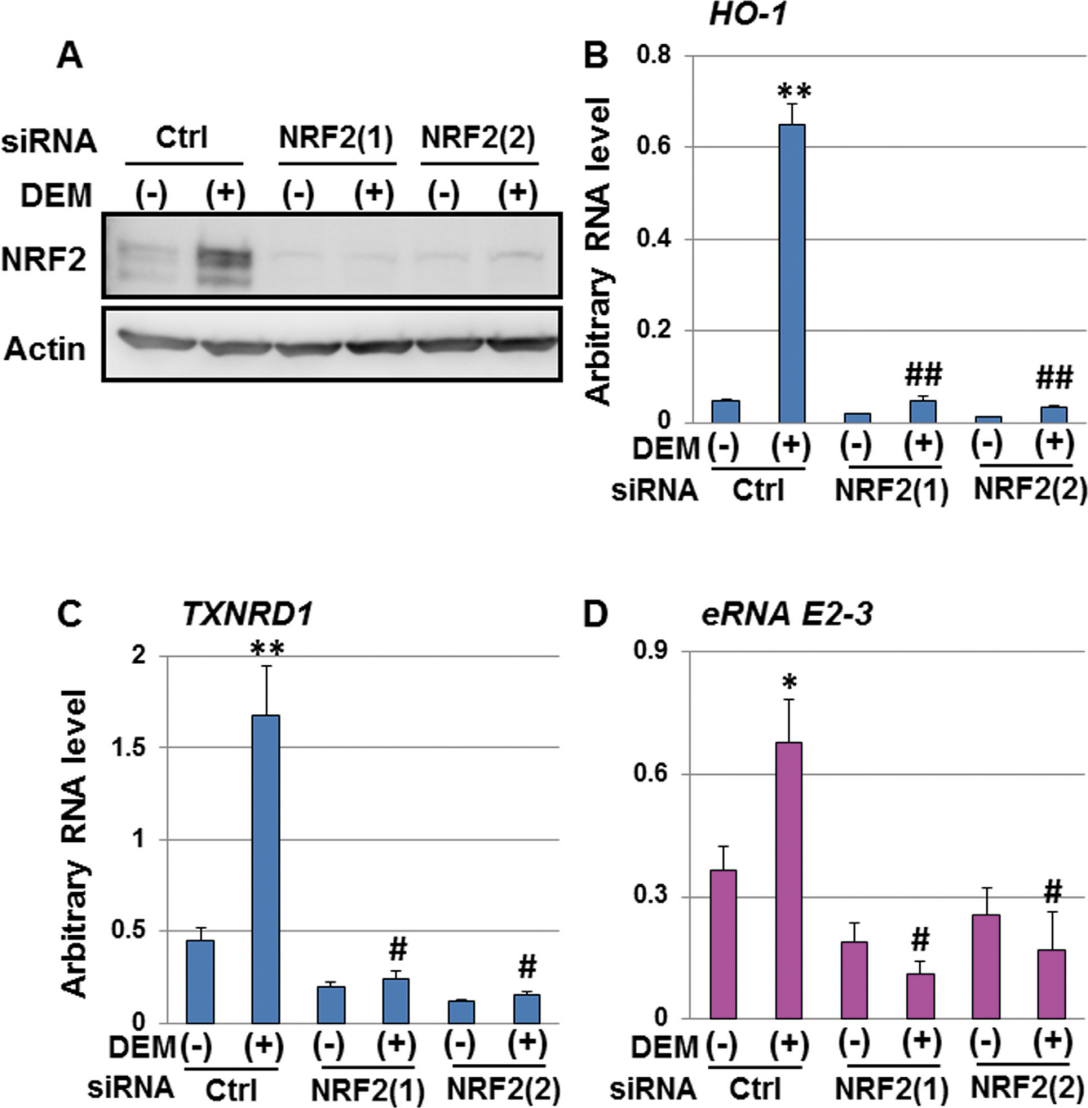
Expression of the *eRNA E2-3* is dependent upon NRF2. (A) The effect of *NRF2*-KD on NRF2 protein expression. HeLa cells were transfected with control siRNA (Ctrl) or *NRF2*-specific siRNAs (NRF2) and exposed to 100 μM DEM for 6 h, and whole-cell lysates were then prepared. Whole-cell lysates were separated by SDS-PAGE, and NRF2 protein expression was analyzed by immunoblotting using an NRF2-specific antibody (Santa Cruz, sc-13032). Actin was used as a loading control. The effect of *NRF2*-KD on DEM-responsive *HO-1* (B) and *TXNRD1* expression (C). *HO-1* and *TXNRD1* expression was analyzed by real-time RT-PCR using the TaqMan^®^ Gene Expression Assay system. (D) The effect of *NRF2*-KD on DEM-responsive *eRNA E2-3* expression. The arbitrary RNA level was measured by real-time RT-PCR using specific primers and Universal Probe Library Probe. Each value was normalized to cyclophilin A gene expression, and the arbitrary RNA level was expressed as the mean ± SEM of three independent assays. *P < 0.05; **P < 0.01 (two-tailed unpaired Student's *t*-test), #*P* < 0.05; ##*P* < 0.01 compared to the value of the control siRNA with 100 μM DEM for 6 h (Ctrl (+)) (one-way ANOVA followed by a Dunnett's *post hoc* test for multiple parameter comparisons).

Because our results showed that Pol II binding to the E2 enhancer showed increased trend in response to DEM (Figure 1), we further focused on the role of *eRNA E2-3* in *HO-1* regulation. We investigated the contribution of NRF2 to the expression of *eRNA E2-3* using two *NRF2*-specific siRNAs in HeLa cells. We confirmed that DEM-induced NRF2 protein expression was repressed by both *NRF2* siRNAs compared with the control siRNA (Figure 5A). The expression of *HO-1* and *TXNRD1* was significantly attenuated by*NRF2* knockdown (KD) as judged by real-time RT-PCR (Figure 5B and C). We found that DEM-induced *eRNA E2-3* expression was significantly down-regulated in *NRF2*-KD cells (Figure 5D).

**Figure 6.**
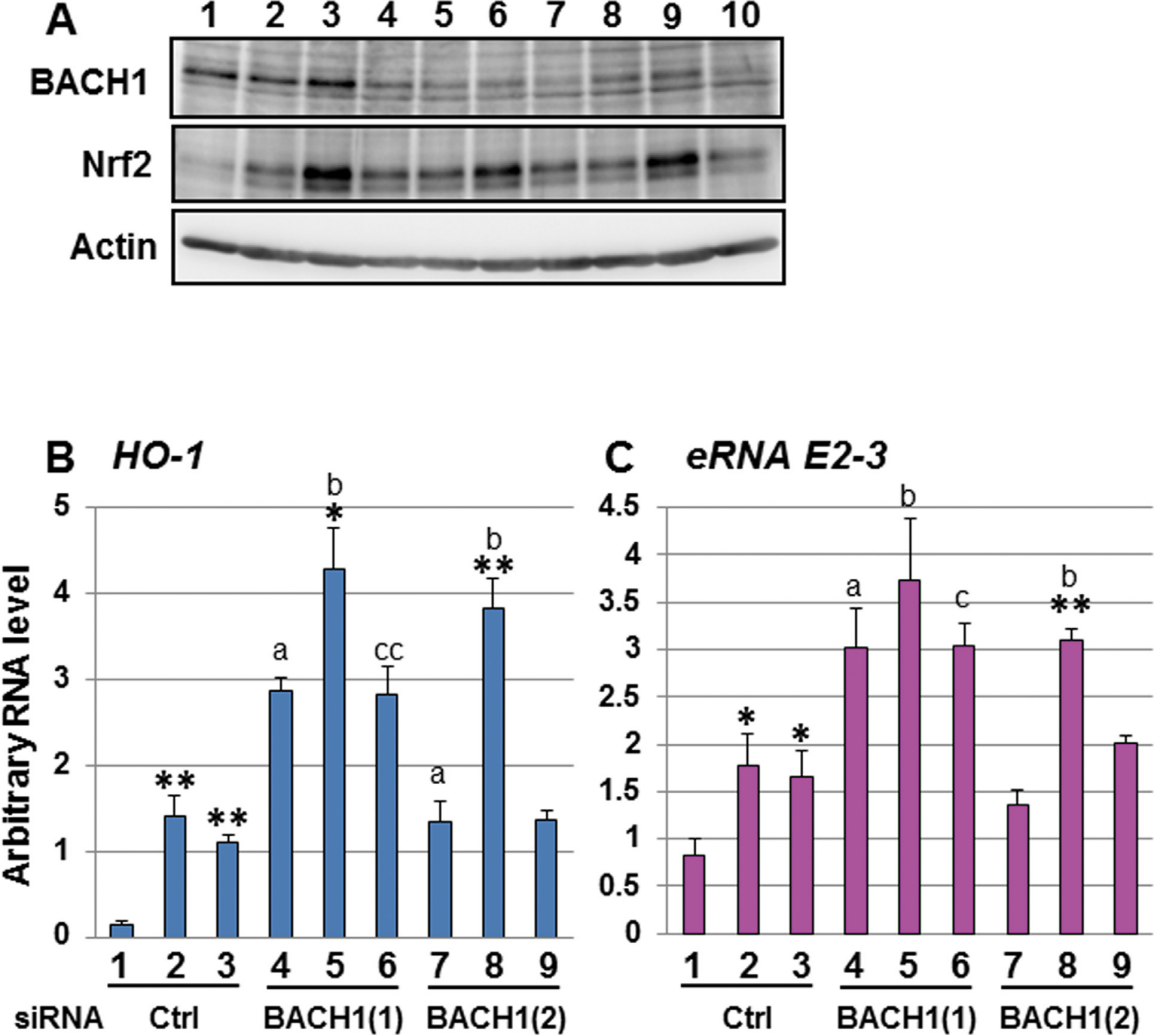
Expression of the *eRNA E2-3* is repressed by BACH1. (A) The effect of *BACH1*-KD on BACH1 and NRF2 protein expression. HeLa cells were transfected with either control siRNA (Ctrl, lanes 2–4) or *BACH1*-specific siRNA (BACH1(1) or BACH1(2) (lanes 5–7 or lanes 8–10). The cells were then treated either with dimethyl sulfoxide (DMSO) (lane 2, 5 and 8), 100 μM DEM (lane 3, 6 and 9) or 20 μM Hemin (lane 4, 7 and 10) for 6 h and whole-cell lysates were prepared. Whole-cell lysates were separated by SDS-PAGE, and BACH1 or NRF2 protein expression were analyzed by immunoblotting using a BACH1-specific antibody (Santa Cruz, sc-14700) or an NRF2-specific antibody (Santa Cruz, sc-13032). Whole-cell lysates from human BACH1 expression plasmid-transfected HeLa cells were used as a positive control for BACH1 protein expression (lane 1). (B and C) The effect of *BACH1*-KD on *HO-1* (B) and *eRNA E2-3* (C). HeLa cells were transfected by control siRNA (Ctrl, lanes 1–3), *BACH1* siRNA (1) (BACH1(1), lanes 4–6) or *BACH1* siRNA (2) (BACH1(2), lanes 7–9). The cells were then exposed to either DMSO (lanes 1, 4 and 7), 100 μM DEM (lanes 2, 5 and 8) or 20 μM Hemin (lanes 3, 6 and 9) for 6 h, and total RNA samples were isolated. *HO-1* expression was analyzed by real-time RT-PCR using the TaqMan^®^ Gene Expression Assay system. The *eRNA E2-3* level was measured by real-time RT-PCR using specific primers and Universal Probe Library Probe. Each value was normalized to cyclophilin A gene expression and the arbitrary RNA level was expressed as the mean ± SEM of three independent assays. *P < 0.05; **P < 0.01 (two-tailed unpaired Student's *t*-test) compared to DMSO (lanes 1, 4 or 7), a: *P* < 0.05, compared to the value of the control siRNA with DMSO (lane 1); b: *P* < 0.05, compared to the value of the control siRNA with 100 μM DEM, 6 h (lane 2); c: *P* < 0.05; cc: *P* < 0.01, compared to the value of the control siRNA with 20 μM Hemin, 6 h (lane 3) (one-way ANOVA followed by a Dunnett's *post hoc* test for multiple parameter comparisons).

*HO-1* induction is regulated by the balance between NRF2 activation and BACH1 repression. Therefore, we examined whether BACH1 regulates the expression of *eRNA E2-3*. We transiently transfected two independent siRNAs against the BACH1 gene (*BACH1*) into HeLa cells and analyzed the RNA levels of the *eRNA E2-3* by real-time RT-PCR. *BACH1*-KD was confirmed by immunoblotting using the BACH1-specific antibody (Figure 6A). The basal level of *HO-1* expression was enhanced in *BACH1-*KD cells compared to control cells (Figure 6B). DEM-induced, but not Hemin-induced, *HO-1* expression was further up-regulated by *BACH1*-KD compared to the control-KD (Figure 6B). Note that NRF2 protein expression was induced by DEM, but not by Hemin in HeLa cells (Figure 6A). We detected a similar trend for *eRNA E2-3* expression in *BACH1*-KD cells compared to control cells (Figure 6C). Taken together, these results indicate that the expression of *eRNA E2-3* in response to DEM is induced by NRF2, while BACH1 negatively regulates *eRNA E2-3* expression in response to DEM.

### *eRNA E2-3* selectively regulates DEM-induced *HO-1* expression

**Figure 7.**
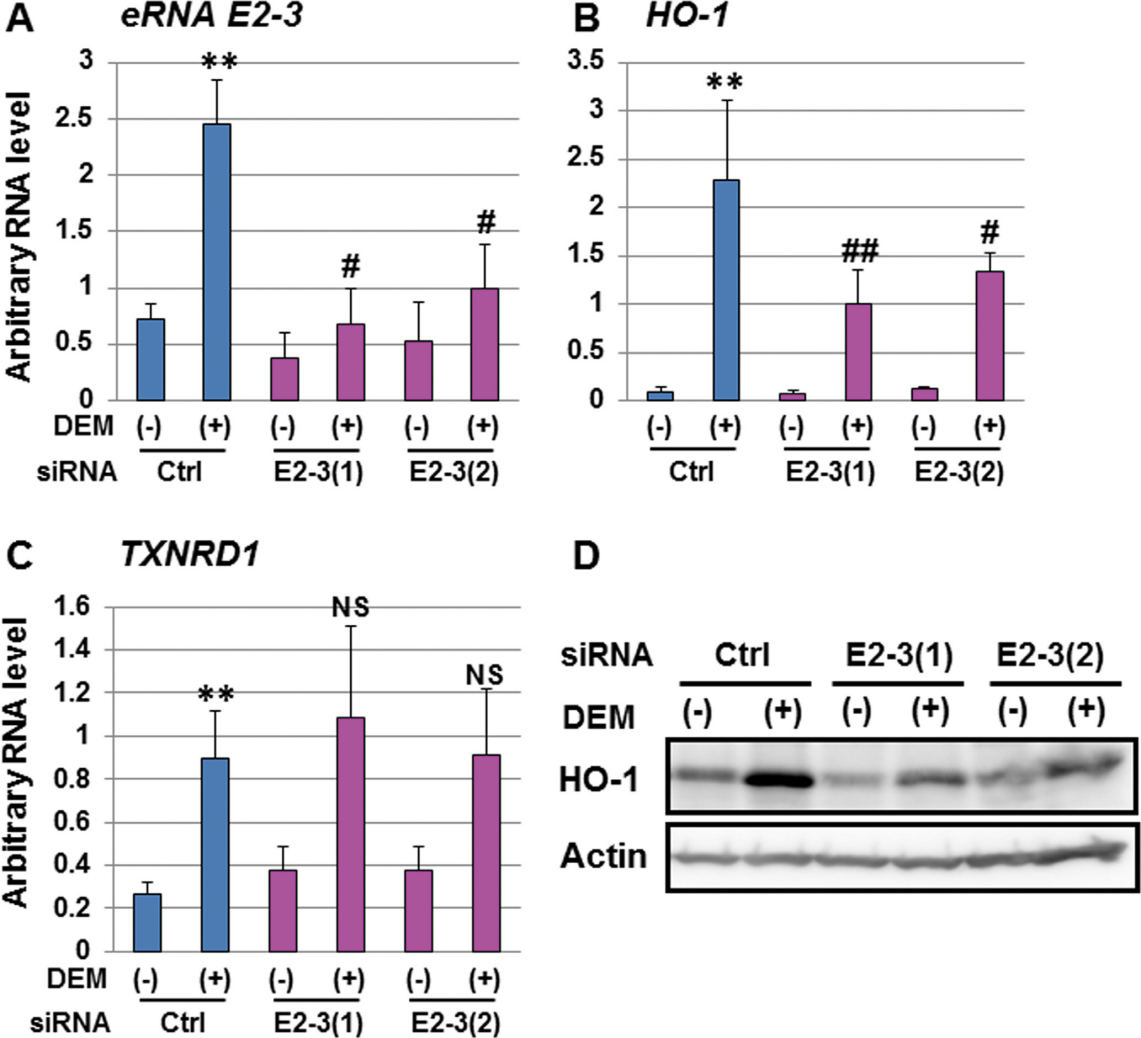
*eRNA E2-3* selectively regulates *HO-1* induction. (A) The effect of *eRNA E2-3*-KD on *eRNA E2-3* expression. HeLa cells were transfected with control siRNA (Ctrl) or two siRNAs against *eRNA E2-3* (E2-3(1) or E2-3(2)) and were untreated (-) or exposed to 100 μM DEM for 6 h (+). The *eRNA E2-3* level was analyzed by real-time RT-PCR using specific primers and Universal Probe Library Probe. (B and C) The effect of *eRNA E2-3*-KD on the expression of *HO-1* (B) and *TXNRD1* (C). The expression of *HO-1* and *TXNRD1* was analyzed by real-time RT-PCR using the TaqMan^®^ Gene Expression Assay system. Each value was normalized to cyclophilin A gene expression, and the arbitrary RNA level was expressed as the mean ± SEM of four independent assays. **P < 0.01 (two-tailed unpaired Student's *t*-test) compared to control siRNA without DEM (Ctrl (-)). #: *P* < 0.05; ##: *P* < 0.01; NS, not significant compared to the value of control siRNA with 100 μM DEM for 6 h (Ctrl (+)) (one-way ANOVA followed by a Dunnett's *post hoc* test for multiple parameter comparisons). (D) The effect of *eRNA E2-3-*KD on HO-1 protein expression. Whole-cell lysates were separated by SDS-PAGE and HO-1 protein expression was analyzed by immunoblotting using a HO-1-specific antibody (Abcam, ab68477). Actin was used as a loading control (Sigma-Aldrich, A1978).

It has been reported that the production of eRNAs is associated with the expression of the adjacent coding mRNA (37). To clarify the role of *eRNA E2-3* in NRF2-dependent *HO-1* induction, we performed KD analysis of *eRNA E2-3* using two specific siRNAs. We confirmed that the siRNA actually decreased the level of *eRNA E2-3* by real-time RT-PCR (Figure 7A). Note that siRNAs against the *eRNA E2-3* region also reduced the expression of *eRNA E2-2* and *vice versa*, supporting the result that *eRNA E2-2* and *eRNA E2-3* are overlapping (Supplementary Methods S1, Supplementary Figures S3 and S9).

Interestingly, *HO-1* induction by DEM was significantly reduced in both *eRNA E2-3*-KD cells compared to control cells (Figure 7B and Supplementary Figure S9D). On the other hand, the DEM-inducible expression of *TXNRD1* in *eRNA E2-3*-KD cells was comparable to that in control siRNA-KD cells (Figure 7C and Supplementary Figure S9E). Furthermore, HO-1 protein expression was also decreased in *eRNA E2-3*-KD cells compared to control cells (Figure 7D). We also confirmed that both of *eRNA E2-3* siRNAs significantly reduced DEM-induced *HO-1* expression compared to control siRNA transfected cells in HaCaT (Supplementary Figure S10).

**Figure 8.**
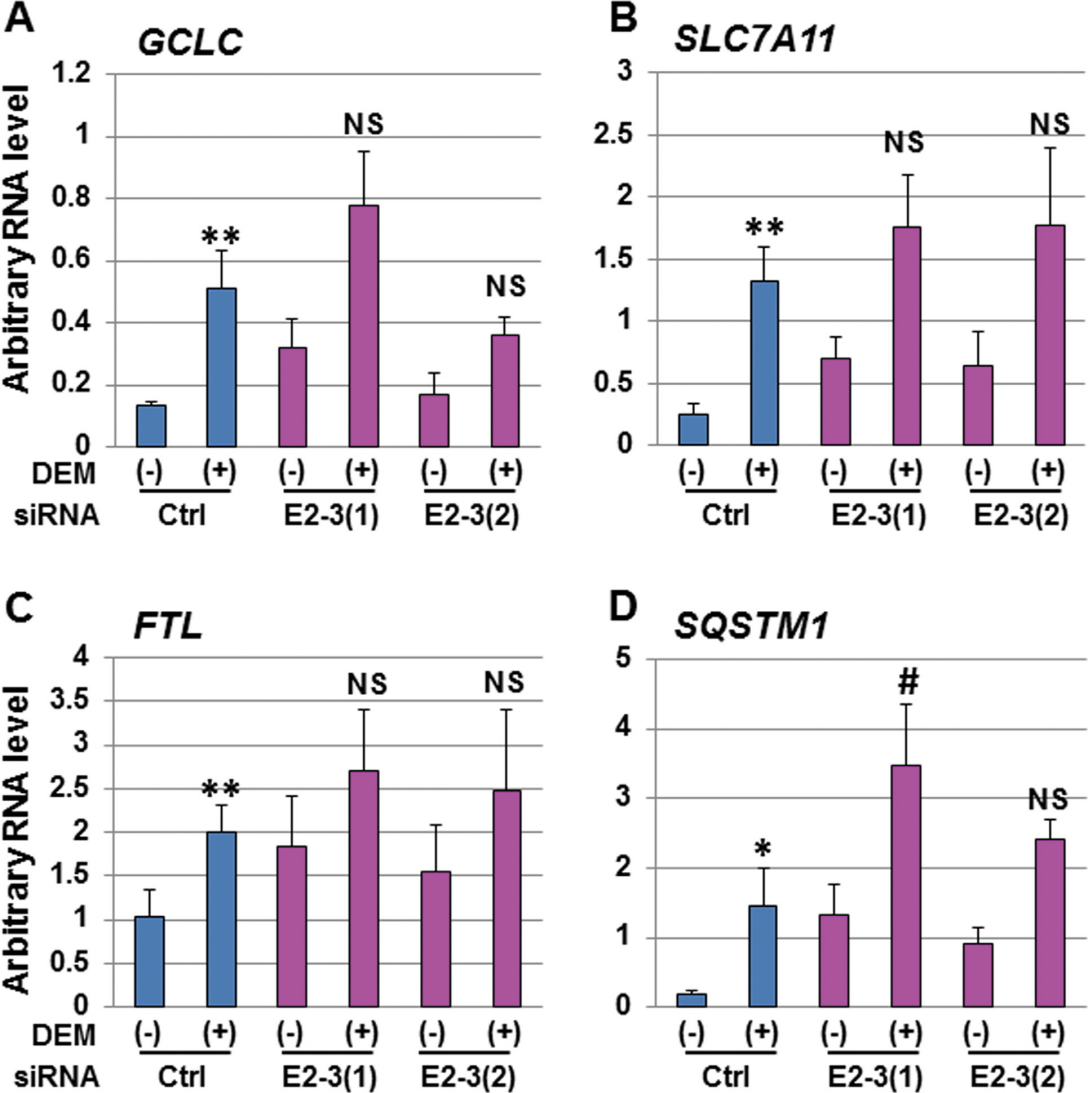
The expression of NRF2-regulated genes in *eRNA E2-3*-KD cells. The effect of *eRNA E2-3*-KD on NRF2-regulated genes, including glutamate-cysteine ligase catalytic subunit (*GCLC*) (A), solute carrier family 7 member 11 (*SLC7A11*) (anionic amino acid transporter light chain, x_c_^−^ system) (B), ferritin light polypeptide (*FTL*) (C) and sequestosome 1 (*SQSTM1*) (D). HeLa cells were transfected with control siRNA (Ctrl) or two siRNAs against *eRNA E2-3* (E2-3(1) or E2-3(2)) and were untreated (-) or exposed to 100 μM DEM for 6 h (+). Total RNA samples were isolated and the RNA levels were measured by real-time RT-PCR. The primers used for real-time RT-PCR are listed in Supplementary Table S1. Each value was normalized to cyclophilin A gene expression and the arbitrary RNA level was expressed as the mean ± SEM of three independent assays. *P < 0.05; **P < 0.01 (two-tailed unpaired Student's *t*-test). #*P* < 0.05; NS, not significant compared to the value of control siRNA with 100 μM DEM for 6 h (Ctrl (+)) (one-way ANOVA followed by a Dunnett's *post hoc* test for multiple parameter comparisons).

To further investigate how *eRNA E2-3* affects the expression of NRF2-regulated genes, we examined the expression of glutamate-cysteine ligase catalytic subunit (*GCLC*), solute carrier family 7 member 11 (*SLC7A11*), ferritin light polypeptide (*FTL*) and sequestosome 1 (*SQSTM1*) in *eRNA E2-3*-KD cells. We observed that the RNA levels of other NRF2-regulated genes were not significantly affected by *eRNA E2-3-*KD compared to the control siRNA (Figure 8). Collectively, these results indicate that *eRNA E2-3* selectively regulates DEM-induced *HO-1* expression.

### *eRNA E2-3* modulates *HO-1* induction by enhancing Pol II binding to the promoter and enhancer regions of *HO-1*

**Figure 9.**
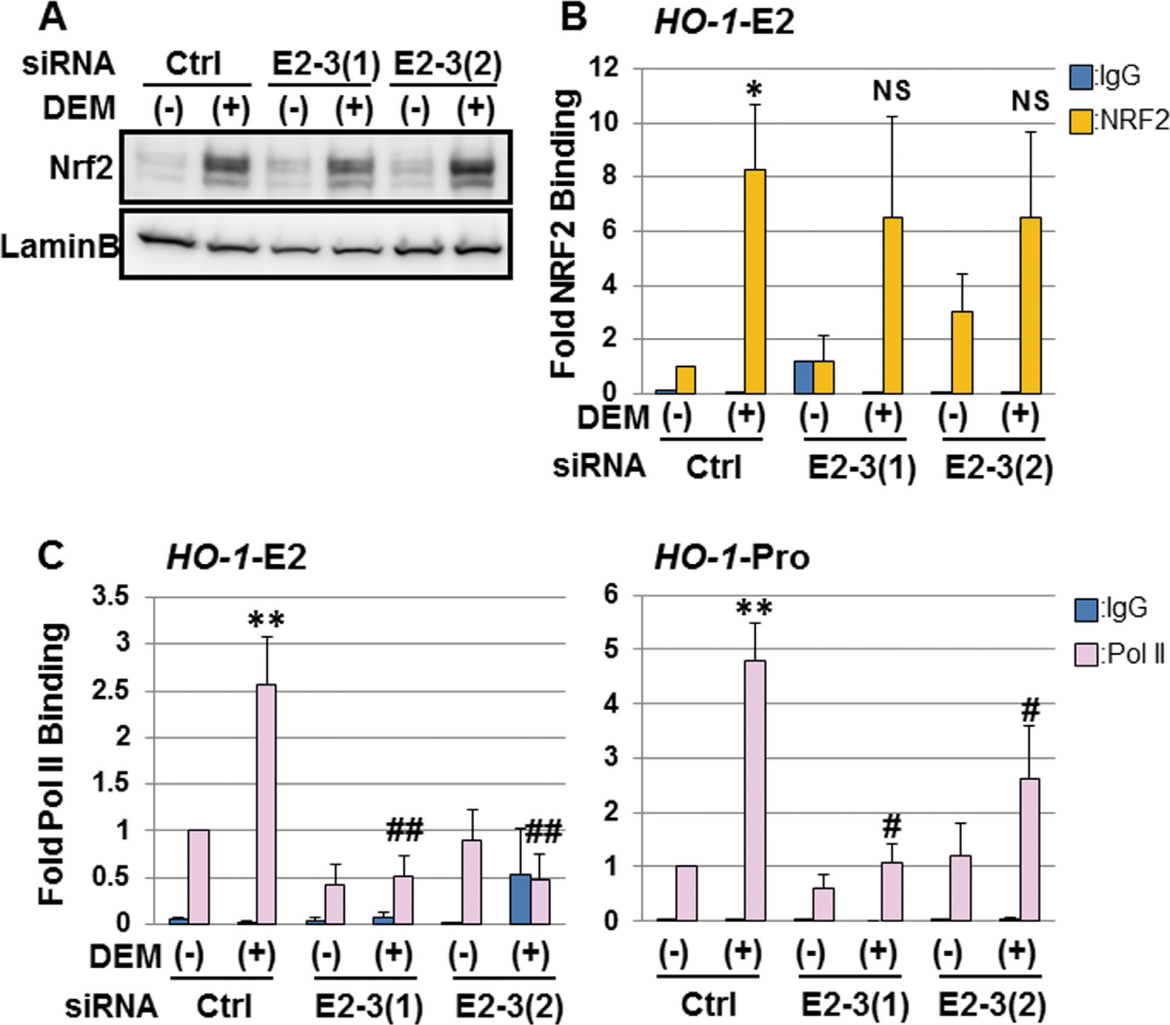
*eRNA E2-3* modulates Pol II binding to the promoter and enhancer regions of the *HO-1* region. (A) NRF2 accumulation in *eRNA E2-3*-KD cells. HeLa cells were transfected with control siRNA (Ctrl) or siRNAs against *eRNA E2-3* (E2-3(1) or E2-3(2)) and were untreated (-) or exposed to 100 μM DEM for 6 h (+). Nuclear extracts were separated by SDS-PAGE and the NRF2 protein level was analyzed by immunoblotting using a NRF2-specific antibody (Santa Cruz, sc-13032). Lamin B was used as a loading control (Santa Cruz, sc-6217). (B) NRF2 binding to the *HO-1* E2 enhancer (*HO-1*-E2) in *eRNA E2-3*-KD cells. siRNA-transfected HeLa cells were untreated (−) or exposed to 100 μM DEM for 3 h (+), and cell lysates were prepared. ChIP assays were performed using an anti-NRF2 antibody (Santa Cruz, sc-13032) (yellow bars). Normal mouse IgG was used as a negative control (blue bars). (C) Pol II binding to *HO-1* E2 enhancer (*HO-1*-E2) and *HO-1* promoter (*HO-1*-Pro) regions in *eRNA E2-3*-KD cells. ChIP assays were performed using an anti-Pol II antibody (Millipore, clone CTD4H8) (pink bars). Normal mouse IgG was used as a negative control (blue bars). Binding of NRF2 or Pol II was measured by real-time PCR using specific primers against the regions shown in Figure 1(A). The value for control siRNA-transfected cells without DEM (Ctrl (-)) was arbitrarily set as 1, and the fold binding was expressed as the mean ± SEM of three independent assays. *P < 0.05; **P < 0.01 (two-tailed unpaired Student's *t*-test) compared to the value of control siRNA-untreated cells (Ctrl (−)), #*P* < 0.05; NS, not significant compared to the value of control siRNA-treated cells with 100 μM DEM for 3 h (Ctrl (+)) (one-way ANOVA followed by a Dunnett's *post hoc* test for multiple parameter comparisons).

NRF2 activation is a critical step for NRF2-dependent *HO-1* induction (33,38). We demonstrated by immunoblot analysis that DEM-induced NRF2 nuclear accumulation in *eRNA E2-3*-KD cells was comparable to control cells (Figure 9A). Consistent with this observation, ChIP analysis showed that NRF2 binding to the E2 enhancer in *eRNA E2-3*-KD cells was not significantly changed compared to control cells (Figure 9B). On the other hand, the induction of Pol II binding to the promoter and E2 enhancer was significantly decreased in *eRNA E2-3*-KD cells compared to control cells (Figure 9C). These results indicate that *eRNA E2-3* modulates *HO-1* induction by enhancing Pol II binding to the promoter and enhancer regions of *HO-1* without affecting the binding of NRF2 to the enhancer.

## DISCUSSION

**Figure 10.**
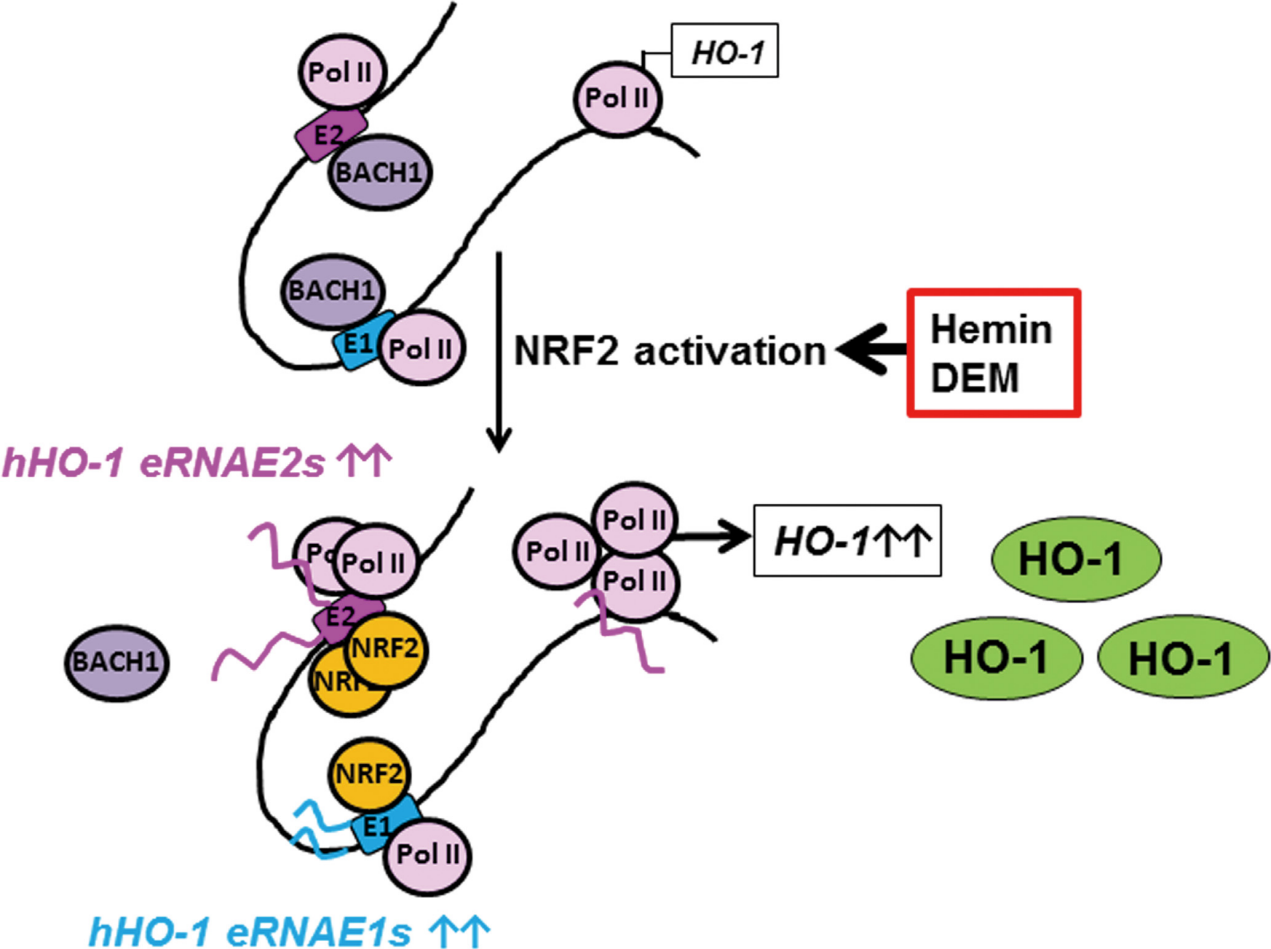
Schematic model of the function of *hHO-1 eRNAs* in *HO-1* induction in response to NRF2 activation. A hypothetical molecular mechanism of *hHO-1 eRNAs*-mediated NRF2-dependent *HO-1* induction is shown. The details are described in the text.

In this study, we showed for the first time that the regions adjacent to the human *HO-1* enhancers were actively transcribed and that some of the transcripts were inducible in response to DEM. In addition, we demonstrated that *eRNA E2-3* was required for enhancer- and NRF2-mediated human *HO-1* induction (Figure 10). In the resting state, *HO-1* expression was repressed by BACH1 (Figure 6) (16), although some Pol II was already bound to the promoter and enhancer regions of *HO-1* (Figure 1C). In response to environmental stimuli, NRF2 accumulated in the nucleus and bound to *HO-1* enhancer regions (Figure 9A and B). NRF2-dependent *eRNA E2-3* transcription led to the inducible recruitment of Pol II to the E2 enhancer and the promoter of *HO-1* (Figure 9C). We surmise that increased Pol II binding stimulates *HO-1* transcription.

We found that multiple and bi-directional enhancer RNA signals are generated from human *HO-1* enhancer regions (Figure 2 and Supplementary Figures S1–S3). Because these transcripts are more than 200 nucleotides in length, the *hHO-1 eRNAs* are classified as lncRNAs. Although we performed northern blot analysis to determine the full-length structure of the E2-3 and E1-4 regions, we failed to detect specific RNA signals corresponding to them (data not shown). We have also carried out 3′ RACE analysis of the E2-3 region using Oligo-dT primer for RT reactions. However, we unfortunately obtained 3′ RACE products derived from A-rich sequences that exist in the E2-3 region itself (data not shown). Next, we conducted 3′ primer walking analyses using a series of primers on the upstream region of human *HO-1*. As a result, we successfully obtained a 1.3-kb spliced RT-PCR signal of *eRNA E2-3* and named it *hHO-1 eRNA E2_L* (Supplementary Figures S6 and S7). We also detected a RT-PCR product where the reverse primer exists in the putative intron in a semi-quantitative RT-PCR analysis (Supplementary Figure S6, primer set (i)). It is still unknown whether *hHO-1 eRNAs* have a poly A tail or not. Therefore, to further clarify the role of *hHO-1 eRNAs*, it is important to determine the full-length structure of these lncRNAs in a future study.

Although we determined the putative 5′ ends of *eRNA E2-3* and *eRNA E1s* (Supplementary Figures S4 and S5), these 5′ ends were different from those found in CAGE (24). The CAGE data were obtained using a cDNA library constructed from human hepatoma HepG2 cells, while total RNA isolated from HeLa cells was used as the 5′ RACE template. Thus, it is possible that *hHO-1 eRNAs* have cell- or tissue-specific transcriptional start sites. This might be reflected in the expression level of *hHO-1 eRNAs* among human cell lines (Figure 2 and Supplementary Figure S8). In addition, recent FAMTOM 5 data indicated that human *HO-1* enhancers are potential active bi-directional promoters, and we found RNA signals corresponding to *eRNA E2-1* and *eRNA E1s* (35). We also found that transcripts corresponding to *eRNA E2-1* and *eRNA E1s* were detected by Cold Spring Harbor Laboratory long RNA–sequencing data obtained from the UCSC genome browser (36). Those data reveal that the expression profile of *hHO-1 eRNAs* varies among cell types and tissues. Thus, by analyzing the relationship between *HO-1* expression and *hHO-1 eRNAs* in various tissues, the contribution of *hHO-1 eRNAs* in *HO-1* expression and its physiological significance will be further clarified.

Although the reported human RNA-sequencing data and our observations clearly show the existence of *hHO-1 eRNAs*, ARE elements in two *HO-1* enhancer regions are highly conserved between human and mouse (39). However, the nucleotide sequence adjacent to the *HO-1* enhancers is highly conserved among primates, including chimpanzee and orangutan, but diverges in rodents (Supplementary Figures S4 and S5) (34). Therefore, the regulation of *HO-1* expression by enhancer RNAs in other species should be carefully examined in future experiments.

Our data demonstrated *eRNA E2-3* and *eRNA E1-4* are mainly enriched in the cell nucleus (Figure 3), suggesting that these *hHO-1 eRNAs* work as a ncRNA and regulate nuclear functions, such as transcription. We showed that E2 enhancer-derived *eRNA E2-3* selectively regulates DEM-responsive *HO-1* expression by mediating Pol II recruitment of the *HO-1* locus (Figures 7–9 and Supplementary Figure S10). We previously reported that BRG1, an ATPase subunit of the chromatin-remodeling complex, interacts with NRF2 to selectively modulate *HO-1* induction in response to DEM (21). Additionally, we showed that this selectivity is associated with Z-DNA formation at the human *HO-1* promoter region (23). In this study, we demonstrated that *eRNA E2-3* is specifically involved in the induction of *HO-1* expression in response to DEM and most likely acts by a *cis*-acting mechanism (Figures 7 and 9). Thus, although both BRG1 and *eRNA E2-3* participate in *HO-1* induction, whether there is a direct mechanistic link remains to be clarified in future analyses.

We were interested in the role of *eRNA E2-1* and also investigated the subcellular localization of its RT-PCR signal. We found that *eRNA E2-1* localized to the cell nucleus (data not shown), suggesting that *eRNA E2-1* is involved in *HO-1* expression. Although we attempted a knockdown analysis of *eRNA E2-1*, we failed to obtain any conclusive results. Thus, the function of other *hHO-1 eRNAs*, including *eRNA E2-1*, should be examined in future analyses.

Recent studies have uncovered diverse mechanisms of transcription regulation by eRNAs. For example, eRNAs from tumor suppressor p53-bound enhancer regions are required for p53-dependent enhancer activity and the activation of neighboring genes when tethering Pol II to the promoter (40). In an estrogen-stimulated breast cancer, eRNAs induced by the activation of estrogen receptor α increase the chromatin looping between the enhancer region and the promoter of target genes, and the enhancer-promoter looping is stabilized through the interaction between eRNAs, cohesin and the transcription machinery (41). A recent paper showed that eRNA expressed from the core enhancer element of the MyoD gene facilitates the recruitment of chromatin remodeling factors to the open chromatin state, leading to an increase in Pol II occupancy at the promoter region of the MyoD gene during skeletal muscle differentiation (42). We showed that *eRNA E2-3* regulate *HO-1* induction by modulating Pol II binding to the promoter and enhancer regions of the *HO-1* region (Figure 9). Collectively, these reports and our data suggest that one of the functions of *eRNA E2-3* is the regulation of Pol II binding to a gene promoter, although the precise molecular mechanism for the human *HO-1* locus remains elusive.

Recent genome-wide sequencing studies, such as the ENCODE project, showed that non-coding regions are transcribed and play important roles, including transcription regulation (25). The most recent work reported that a recessive mutation in a distal enhancer region of the PTF1A gene leads to pancreatic agenesis (43), indicating that the non-coding region is a potential determinant of disease. HO-1 plays a central role in protecting against disease, including inflammation, and regulates various cellular processes by producing CO, biliverdin/bilirubin and ferrous ion. Therefore, *hHO-1 eRNAs* may be a hopeful therapeutic target for regulating *HO-1* expression.

## SUPPLEMENTARY DATA

Supplementary Data are available at NAR Online.

SUPPLEMENTARY DATA
