# Supplementary material for: Non-coding RNA derived from the region adjacent to the human HO-1 E2 enhancer selectively regulates HO-1 gene induction by modulating Pol II binding
Source: Nucleic Acids Res. 2014 Nov 17;42(22):13599–614. doi: 10.1093/nar/gku1169 (PMC4267629; doi:10.1093/nar/gku1169)
Supplement: SUPPLEMENTARY DATA [file supp_42_22_13599__index.html]

Non-coding RNA derived from the region adjacent to the human HO-1 E2 enhancer selectively regulates HO-1 gene induction by modulating Pol II binding — SUPPLEMENTARY DATA 

# Non-coding RNA derived from the region adjacent to the human *HO-1* E2 enhancer selectively regulates *HO-1* gene induction by modulating Pol II binding

## SUPPLEMENTARY DATA

**Files in this Data Supplement:**

- SUPPLEMENTARY DATA
