# Supplementary material for: Non-coding RNA derived from the region adjacent to the human HO-1 E2 enhancer selectively regulates HO-1 gene induction by modulating Pol II binding
Source: Nucleic Acids Res. 2014 Nov 17;42(22):13599–614. doi: 10.1093/nar/gku1169 (PMC4267629; doi:10.1093/nar/gku1169)
Supplement: SUPPLEMENTARY DATA [file supp_gku1169_Supplementary_Data_2014.11.14.pdf]

Supplementary Methods S1

Cell culture

Human neuroblastoma SH-SY5Y and immortal human keratinocyte HaCaT cells were cultured in Dulbecco’s Modified Eagle Medium (DMEM) -F12, GlutaMAX (Gibco) or DMEM (Sigma-Aldrich), respectively, and all contained 10% FBS and 100 units/ mL penicillin/ streptomycin. All cells were cultured at 37°C with 5% CO_2 and saturated humidity.

Transcript mapping of human *HO-1* enhancer RNAs

To detect transcripts derived from *HO-1* enhancer regions, we performed transcript mapping. Briefly, cDNA was synthesized using total RNA from DEM-treated HeLa cells as a template with random hexamers, and RT-PCR was performed with primer sets listed in Supplementary Table S1. In RT-PCR analysis, following samples were used as controls: HeLa genomic DNA was used as a positive control of PCR amplification; a reaction without reverse transcriptase was used as a negative control to rule out genome DNA contamination. RT-PCR products were electrophoresed on TAE-agarose gel, visualized by ethidium bromide staining, cloned using a Zero Blunt TOPO PCR Cloning kit (Life Technologies) and the cloned DNA was subjected to the DNA sequence analysis.

Determination of direction of *hHO-1 eRNAs*

To determine the direction of transcripts around the *HO-1* enhancer regions, we performed a strand-specific RT reaction using internal primers followed by cDNA amplification using the primer sets. Briefly, cDNA was synthesized using total RNA from DEM-treated HeLa cells as a template with region specific forward or reverse primers, and subsequent PCR was performed with primer sets listed in Supplementary Table S1. Amplified RT-PCR products were electrophoresed on TAE-agarose gel, visualized by ethidium bromide staining, cloned using a Zero Blunt TOPO PCR Cloning kit (Life Technologies) and the cloned DNA was subjected to the DNA sequence analysis.

5’ Rapid Amplification of cDNA Ends (5’ RACE)

5’ RACE analysis was carried out using a Gene Racer Kit (Life Technologies) according to the manufacturer’s protocol. Briefly, total RNA from DEM-treated HeLa cells was isolated, dephosphorylated and 5’ Cap structure was removed. Then, the GeneRacer RNA oligo was ligated to de-capped 5’ end of RNA and reverse transcription

was done with random hexamers. To amplify 5' RACE product, RT-PCR was performed using the GeneRacer 5' primer and reverse region specific primers listed in Supplementary Table S1. Subsequently, nested PCR was performed using the GeneRacer 5' Nested primer and reverse region specific primers. Nested 5' RACE PCR products were electrophoresed on TAE-agarose gel, visualized by ethidium bromide staining and sub-cloned using a Zero Blunt TOPO PCR Cloning kit. The DNA sequence of the inserts in isolated clones was validated by DNA sequencing. To determine 5' ends, we analyzed DNA sequences of the junction where the GeneRacer RNA oligo was ligated.

siRNA

Stealth siRNAs against *eRNA E2-1 and eRNA E2-2* were synthesized by Invitrogen with the following sequences: *eRNA E2-1* (1): 5'- AGU UGA UAC UCA CCG GGU CCC UUA A -3', *eRNA E2-1* (2): 5'- CAC AGG CUC GGC AGC ACC GUG GGA A-3'. *eRNA E2-2* (1): 5'- CCU UUA GAG CUU AGA GAG UCG AAG A-3', *eRNA E2-2* (2): 5'- UGA GUC ACG GUC CCG AGG UCU AUU U-3'. A stealth control siRNA was obtained from Invitrogen. Then, siRNA (final 20 nM) was transfected to cultured cells using the Lipofectamine RNAi MAX reagent according to the manufacturer's protocol (Life Technologies). Twenty-four hours after transfection, the cells were sub-cultured and exposed to 100 μM DEM for the appropriate time.

Supplemenatary Table S1. Primers used in this study

Experiments	Primer names	5'-3' sequences	Purposes
Tanscription mapping, RT-PCR	map_E2_fwd_1	CGG ATG TGG TAT AAT TAC AGC TGT	transcript mapping E2-1, strand specific cDNA synthesis and PCR
	map_E2_rev_1	CTA GTA GTT GAT ACT CAC CGG GT	transcript mapping E2-1, strand specific cDNA synthesis and PCR
	map_E2_fwd_2	CAG TTC TCA CTT CTG CTC ACT TCT	transcript mapping E2-2, strand specific cDNA synthesis
	map_E2_rev_2	GAT TAA ACC TGG AGC AGC TGG AAC TCT	transcript mapping E2-2, strand specific cDNA synthesis
	E2 Forward_1	GTC TGG GGC CTG AAT CCT A	transcript mapping E2-3, strand specific cDNA synthesis, E2-2, E2-3 overlap detection, UPL_realtime PCR for eRNA E2-3 with universal probe library probe #17
	5' RACE E2 Reverse_1	TGC ATT GCA TTC ACT TGT CCT GCC TCT	transcript mapping E2-3, strand specific cDNA synthesis, 5' RACE of E2, 1stPCR, estimation of full-length eRNA E2.
	map_E2_fwd_4	CTT GGG CTG GAA CCT GGA CTG GT	transcript mapping E2-4
	map_E2_rev_4	AAC TGT GAA AAC GTG ACA AGG CAA AGT	transcript mapping E2-4
	map_E1_fwd_1	AAA GCA TCT CCA CAA TCA AGA TAA AG	transcript mapping E1-1
	map_E1_rev_1	CTG AGA TTG CAC CAC TGC ATT CCA	transcript mapping E1-1
	map_E1_fwd_2	GGG TTC AAG CGA TTC TCC TGC CT	transcript mapping E1-2
	map_E1_rev_2	CAG TAA ATA AGG TCA CAG ACG GTG T	transcript mapping E1-2
	map_E1_fwd_3	AGG AGA ATA TCC AGG CAA GGT CT	transcript mapping E1-3, strand specific cDNA synthesis
	hHO-1 E1_Reverse	CAC TGG TGA CTC AGC AAA ATC T	transcript mapping E1-3, strand specific cDNA synthesis, human HO-1 E1 ChIP, transcript mapping, strand specific cDNA synthesis
	map_E1_fwd_4	AGT GAA ACT TCT AGA AAA CGG CAG AAG CCT	transcript mapping E1-4, strand specific cDNA synthesis
	map_E1_rev_4	CCA CAT TCT AAT CAC CAG AAC CTG TGA	transcript mapping E1-4, strand specific cDNA synthesis
Transcript direction, nested PCR	map_E2_fwd_1	CGG ATG TGG TAT AAT TAC AGC TGT	transcript mapping E2-1, strand specific cDNA synthesis and PCR
	map_E2_rev_1	CTA GTA GTT GAT ACT CAC CGG GT	transcript mapping E2-1, strand specific cDNA synthesis and PCR
	map_E2_fwd_2(Nest)	TCT GCT CAC TTC TGG GCT CAC TTA AGC CT	strand specific RT-PCR E2-2 nested primer, E2-2, E2-3 overlap detection
	hHO-1 E2_Reverse	GGC GGT GAC TTA GCG AAA AT	strand specific RT-PCR E2-2 nested primer, human HO-1 E2 ChIP
	map_E2_fwd_3(Nest)	TGA ATC CTA GGG AAG CCA TAG CAG CT	strand specific RT-PCR E2-3 nested primer
	map_E2_rev_3(Nest)	CCT GCC TCT TTG GGT TCC TCA GCC T	strand specific RT-PCR E2-3 nested primer
	map_E1_fwd_3	AGG AGA ATA TCC AGG CAA GGT CT	transcript mapping E1-3, strand specific cDNA synthesis
	hHO-1 E1_Reverse	CAC TGG TGA CTC AGC AAA ATC T	transcript mapping E1-3, strand specific cDNA synthesis, human HO-1 E1 ChIP, transcript mapping, strand specific cDNA synthesis
	map_E1_fwd_4	AGT GAA ACT TCT AGA AAA CGG CAG AAG CCT	transcript mapping E1-4, strand specific cDNA synthesis
	map_E1_rev_4	CCA CAT TCT AAT CAC CAG AAC CTG TGA	transcript mapping E1-4, strand specific cDNA synthesis
E2-2, E2-3 overlap detection	map_E2_fwd_2(Nest)	TCT GCT CAC TTC TGG GCT CAC TTA AGC CT	strand specific RT-PCR E2-2 nested primer, E2-2, E2-3 overlap detection
	eRNA E2_UPL_Reverse	GGC TAG AGG AGG AGT GAG AGG	E2-2, E2-3 overlap detaction, UPL_realtime PCR for eRNA E2-3 with universal probe library probe #17
E1-3, E1-4 overlap detection	hHO-1 E1_Forward	CTG CTG CGT CAT CTT TGG	E1-3, E1-4 overlap detection, human HO-1 E1 ChIP
	5' RACE E1 Reverse_1	GAG GCT TCT GCC GTT TTC TA	E1-3, E1-4 overlap detection, 5' RACE of E1, 1stPCR, Nested PCR, UPL_realtime PCR for eRNA E1-4 with universal probe library probe #51
5'RACE	5' RACE E2 Reverse_1	TGC ATT GCA TTC ACT TGT CCT GCC TCT	transcript mapping E2-3, strand specific cDNA synthesis, 5' RACE of E2, 1stPCR, estimation of full-length eRNA E2.
	5' RACE E2 Reverse_2	AGA GAT GAA GTT TCA CCG T	5' RACE of E2, Nested PCR,
	5' RACE E2-E1 Reverse_1	ACG CCC GGA TAA TTT TGT ATT	5' RACE of E2-E1, 1stPCR, Nested PCR, estimation of full-length eRNA E2
	5' RACE E1 Reverse_1	GAG GCT TCT GCC GTT TTC TA	E1-3, E1-4 overlap detection, 5' RACE of E1, 1stPCR, Nested PCR, UPL_realtime PCR for eRNA E1-4 with universal probe library probe #51
Estimation of full-length eRNA E2	E2 Forward_1	GTC TGG GGC CTG AAT CCT A	transcript mapping E2-3, strand specific cDNA synthesis, E2-2, E2-3 overlap detaction, UPL_realtime PCR for eRNA E2-3 with universal probe library probe #17
	(i) 5' RACE E2 Reverse_1	TGC ATT GCA TTC ACT TGT CCT GCC TCT	transcript mapping E2-3, strand specific cDNA synthesis, 5' RACE of E2, 1stPCR, estimation of full-length eRNA E2.
	(ii) 5' RACE E2-E1 Reverse_1	ACG CCC GGA TAA TTT TGT ATT T	5' RACE of E2-E1, 1stPCR, Nested PCR, estimation of full-length eRNA E2
	(iii) E2 Reverse_3	AGG AAT GCT AAT TTT CTC CTC GTA	estimation of full-length eRNA E2
	(iv) E2 Reverse_4	CTT CAA CTT CCT GCC ATG GAT GAA	estimation of full-length eRNA E2
	(v) E2 Reverse_5	AGA GCT AGG AAG AGC TGG CTT TA	estimation of full-length eRNA E2
	(vi) E2 Reverse_6	TAC ATG CAG ATT TGG ACA CAG AGT AGT	estimation of full-length eRNA E2
	(vii) E2 Reverse_7	TGG AGT GCA GTC CCT GAC ATA TGG T	estimation of full-length eRNA E2
	(viii) E2 Reverse_8	ATC CAT GAC TTC CCA TCT GTG TAC ACT	estimation of full-length eRNA E2
UPL_realtime PCR	E2-1_UPL_Forward	AAA AAG TTC CCA CGG TGC T	UPL_realtime PCR for E2-1 with universal probe library probe #53
	E2-1_UPL_Reverse	CAC CGG GTC CCT TAA CAA A	UPL_realtime PCR for E2-1 with universal probe library probe #53
	E2-2_UPL_Forward	GCT CAC TTC TGG GCT CAC TTA	UPL_realtime PCR for E2-2 with universal probe library probe #66
	E2-2_UPL_Reverse	GAC TCT CTA AGC TCT AAA GGG TGG T	UPL_realtime PCR for E2-2 with universal probe library probe #66
	E2 Forward_1	GTC TGG GGC CTG AAT CCT A	transcript mapping E2-3, strand specific cDNA synthesis, E2-2, E2-3 overlap detection, UPL_realtime PCR for eRNA E2-3 with universal probe library probe #17
	eRNA E2_UPL_Reverse	GGC TAG AGG AGG AGT GAG AGG	E2-2, E2-3 overlap detaction, UPL_realtime PCR for eRNA E2-3 with universal probe library probe #17
	eRNA E1s_UPL_Forward	TGA AAG GGC AGC TTT AAT GG	UPL_realtime PCR for eRNA E1-4 with universal probe library probe #51
	5' RACE E1 Reverse_1	GAG GCT TCT GCC GTT TTC TA	E1-3, E1-4 overlap detection, 5' RACE of E1, 1stPCR, Nested PCR, UPL_realtime PCR for eRNA E1-4 with universal probe library probe #51
SYBR Green_realtime PCR	hSLC7A11_Forward	CCA TGA ACG GTG GTG TGT T	SYBR Green realtime PCR for SLC7A11
	hSLC7A11_Reverse	GAC CCT CTC GAG ACG CAA C	SYBR Green realtime PCR for SLC7A11
	hFTL_Forward	GCT GAA CCA GGC CCT TTT	SYBR Green realtime PCR for FTL
	hFTL_Reverse	TCC AGG AAG TCA CAG AGA TGG	SYBR Green realtime PCR for FTL
	hSQSTM1_Forward	AGC TGC CTT GTA CCC ACA TC	SYBR Green realtime PCR for SQSTM1
	hSQSTM1_Reverse	CAG AGA AGC CCA TGG ACA G	SYBR Green realtime PCR for SQSTM1
ChIP assay	hHO-1 E2_Forward	CCC TGC TGA GTA ATC CTT TCC	human HO-1 E2 ChIP
	hHO-1 E2_Reverse	GGC GGT GAC TTA GCG AAA AT	strand specific RT-PCR E2-2 nested primer, human HO-1 E2 ChIP
	hHO-1 E1_Forward	CTG CTG CGT CAT CTT TGG	E1-3, E1-4 overlap detection, human HO-1 E1 ChIP
	hHO-1 E1_Reverse	CAC TGG TGA CTC AGC AAA ATC T	transcript mapping E1-3, strand specific cDNA synthesis, human HO-1 E1 ChIP, transcript mapping, strand specific cDNA synthesis
	hHO-1 Pro_Forward	GCC AGA AAG TGG GCA TCA G	human HO-1 promoter ChIP
	hHO-1 Pro_Reverse	CTG AGG ACG CTC GAG GGA G	human HO-1 promoter ChIP
	HGB2 Pro_Forward	TGG CTA GGG ATG AAG AAT AAA AGG	human gamma-globin gene promoter ChIP
	HGB2 Pro_Reverse	ATT GAT AAC CTC AGA CGT TCC AGA AG	human gamma-globin gene promoter ChIP

Supplementary Figure S1

Maruyama et al.

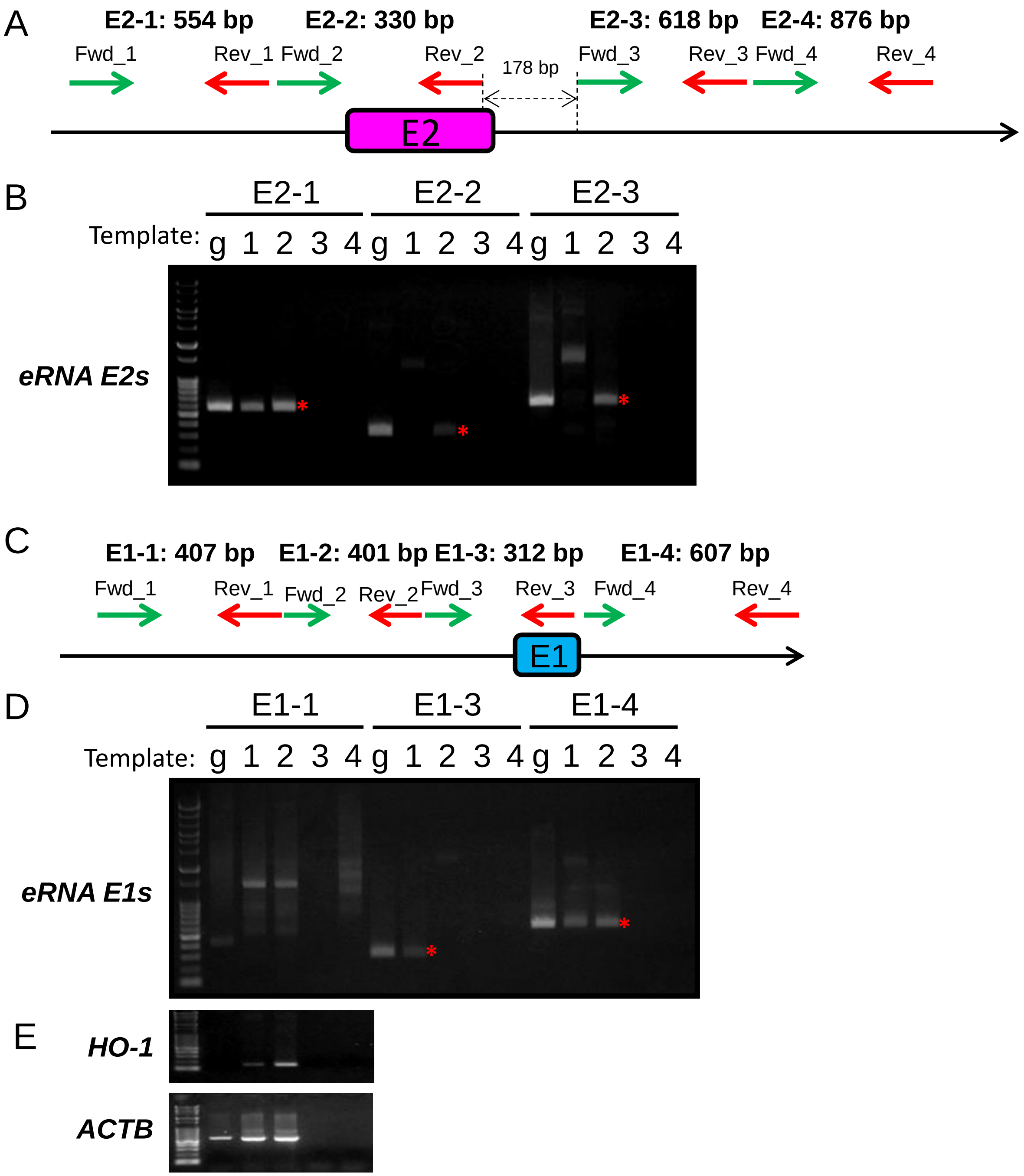

Figure S1 Transcript mapping in *HO-1* enhancer regions and their responsiveness to DEM.
To detect transcripts around the *HO-1* enhancer regions and their DEM response, we performed semi-quantitative RT-PCR. (A) and (C): Primer sets used in the mapping analysis are shown above the schematic presentation of the *HO-1* enhancer regions. Forward primers are indicated in green and reverse primers are in red. The estimated sizes of PCR products are shown above the primer sets. (B), (D) and (E): Ethidium bromide staining of RT-PCR products. We performed RT-PCR using the primer sets indicated in (A) and (C), and the following templates: g: genomic DNA of HeLa cells; 1: RT reaction with random hexamers and DEM-untreated HeLa total RNA; 2: RT reaction with random hexamers and DEM-treated HeLa total RNA; 3: RTase minus reaction with random hexamers and DEM-untreated HeLa total RNA; 4: RTase minus reaction with random hexamers and DEM-treated HeLa total RNA. Amplified RT-PCR products (asterisks) were subcloned using a ZeroBlunt TOPO PCR cloning Kit and the DNA sequence was analyzed. Note that the E2-4 PCR product was detected using genomic DNA as a template, but not using the RT reaction performed with random hexamer and DEM-treated HeLa total RNA (data not shown). On the other hand, E1-2 PCR product was not amplified even when using genomic DNA as a template (data not shown).

Supplementary Figure S2

Maruyama et al.

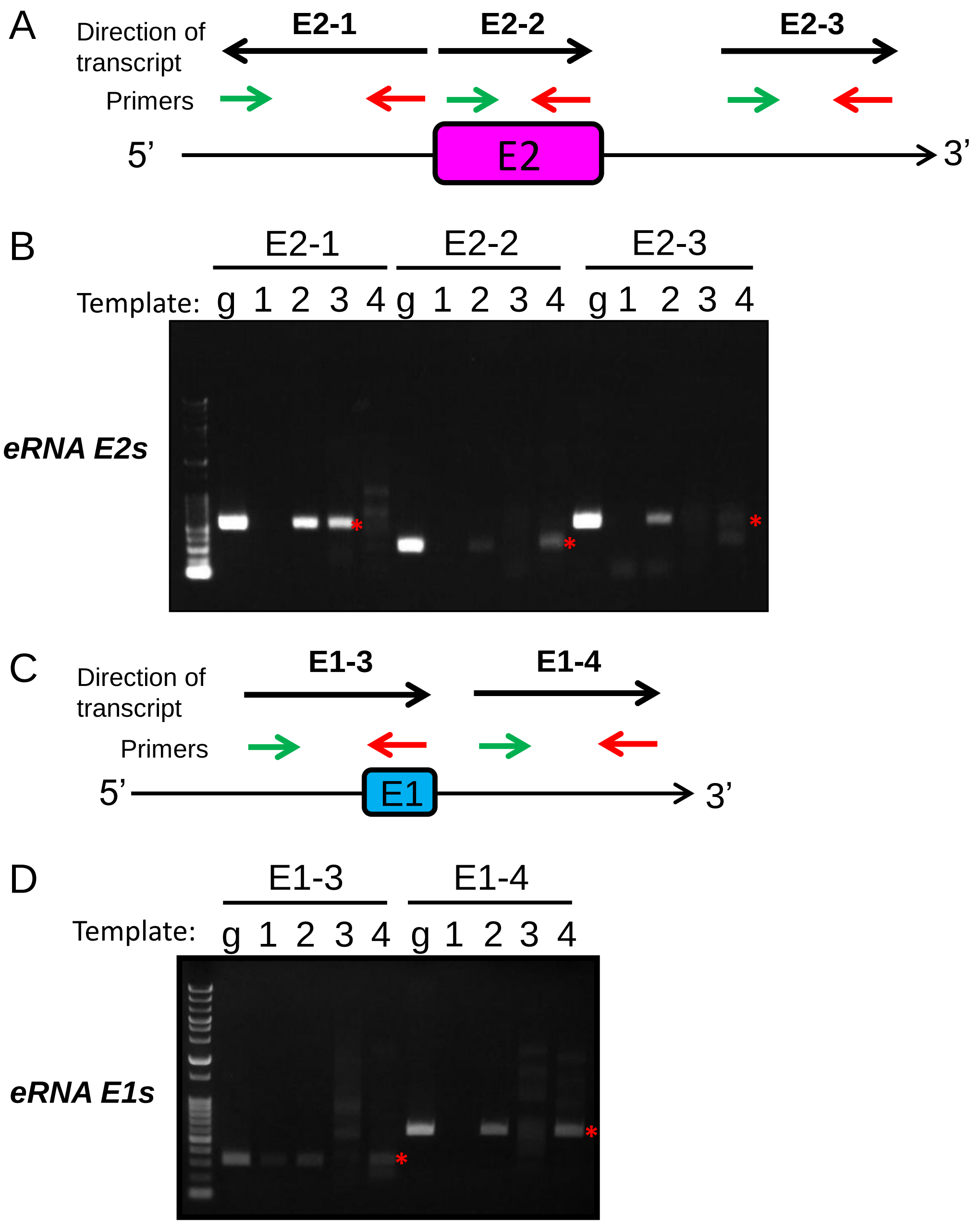

Figure S2 Determination of transcript direction in *HO-1* enhancer regions by strand-specific RT-PCR. To determine the direction of transcripts that were detected around the *HO-1* enhancers, strand-specific RT-PCR was performed using strand-specific gene internal primers and total RNA from DEM-treated HeLa cells. (A) and (C): Primer sets used in RT-PCR analysis are shown above the schematic presentation of the *HO-1* enhancer regions. Forward primers are indicated in green and reverse primers in red. The direction of the transcripts was analyzed by strand-specific RT-PCR reactions. The direction of the transcript determined by the experiments in (B) and (D) is indicated by black arrow in (A) and (C), respectively. (B) and (D): Ethidium bromide staining of RT-PCR products. We performed RT-PCR using the following templates: g: genomic DNA of HeLa cells; 1: RTase minus reaction with random hexamers and DEM-treated HeLa total RNA; 2: RT reaction with random hexamers and DEM-treated HeLa total RNA; 3: RT reaction with a 3'end-directed cDNA synthesis primer and DEM-treated HeLa total RNA ; 4: RT reaction with a 5'end-directed cDNA synthesis primer and DEM-treated HeLa total RNA . Amplified RT-PCR products (asterisks) were subcloned using a ZeroBlunt TOPO PCR cloning Kit and the DNA sequence was analyzed.

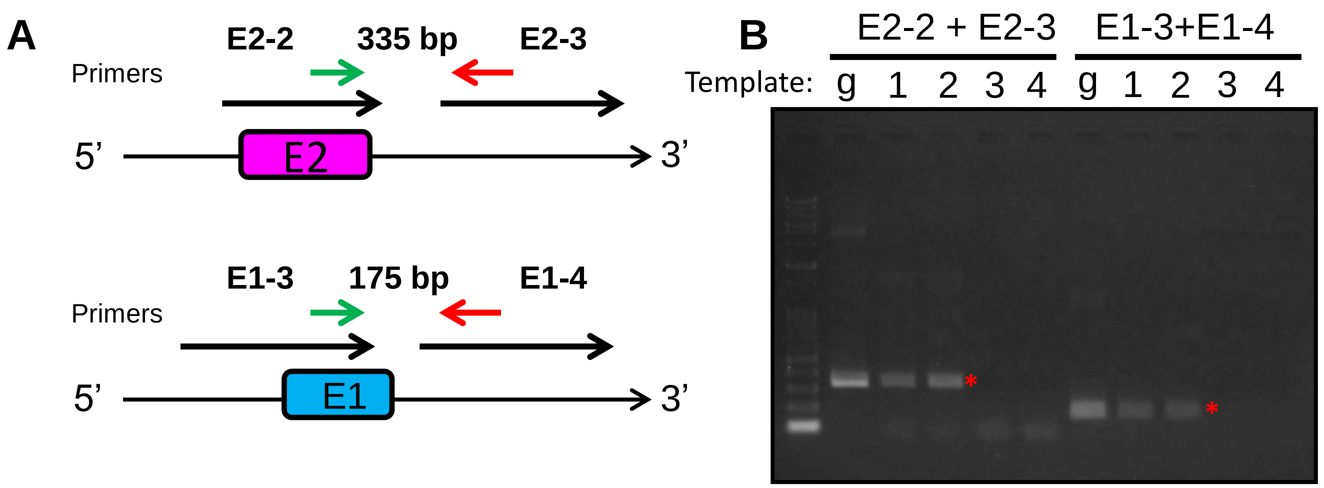

Figure S3 Detection of overlapping RT-PCR signals in the *HO-1* enhancer region.
(A) Previously determined RT-PCR signals and primer sets used in the RT-PCR analysis are shown above the schematic representation of the *HO-1* enhancer regions. Forward primers are indicated in green and reverse primers in red. The directions of the previously determined RT-PCR signals are indicated by black arrow. (B) Ethidium bromide staining of RT-PCR products. We performed RT-PCR using the following templates: g: genomic DNA of HeLa cells; 1: RT reaction with random hexamers and DEM-untreated HeLa total RNA; 2: RT reaction with random hexamers and DEM-treated HeLa total RNA; 3: RTase minus with random hexamers and DEM-untreated HeLa total RNA; 4 : RTase minus reaction with random hexamers and DEM-treated HeLa total RNA. Amplified PT-PCR products (asterisks) were subcloned using a ZeroBlunt TOPO PCR cloning Kit and the DNA sequences were analyzed. Map E2_fwd_2(Nest) and eRNA E2_UPL_Reverse primers were used to amplify the overlapping region between E2-2 and E2-3, and hHO-1 E1_Forward and 5' RACE E1 Reverse_1 primers were used to amplify the overlapping region between E1-3 and E1-4.

Supplementary Figure S4

Maruyama et al.

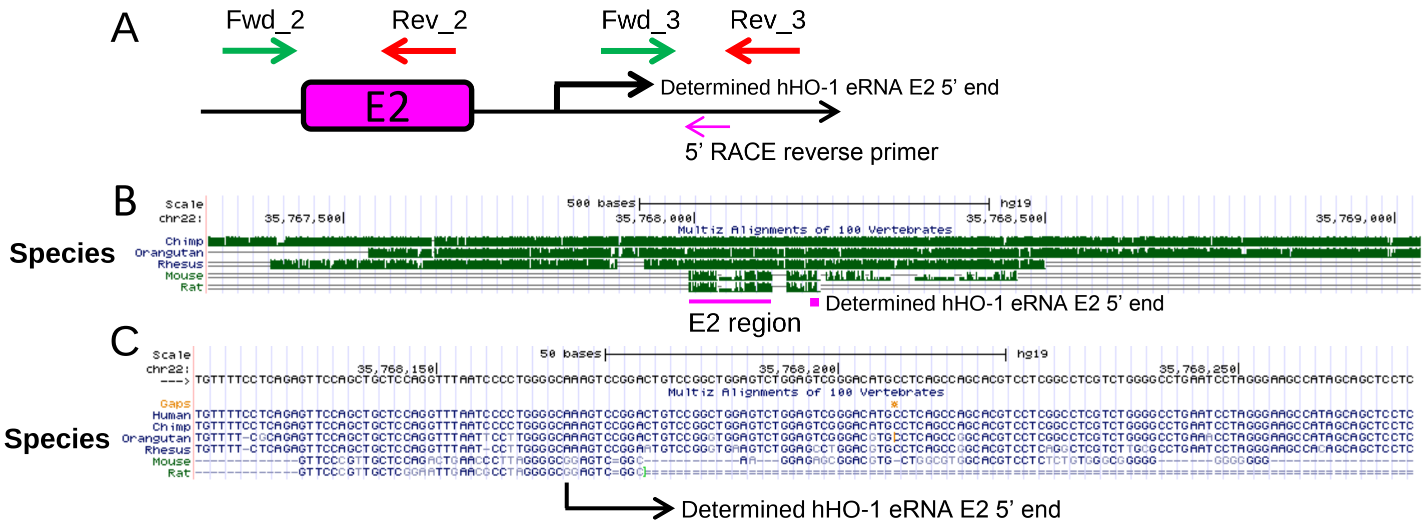

D

AGTATCAACTACTAGGCAGTTCTCACTTCTGCTCACTTCTGGGCTCACTTAAGCCTACCAGCA

Fwd_2

GCCCTGAAGGCTGTTAACCACCCTTTAGAGCTTAGAGAGTCGAAGAGGCAGGGGCCAGGTCCT
AAAGAAAGGCACACTGTCCCCAGAGCCTGGGGCGCGATGCCACCCGCCCCCCCCCCCCCCGCC
CAGGCGTACCCCCCCTTACCCCGCCCCCCACCCGCTCGCCGCGCCCAGCCCATCTGGCGCCGC
TCTGCCCCTGCTGAGTAATCCTTTCCCGAGCCACGTGGCCGTGTTTTTCCTGCTGAGTCACGG
TCCCGAGGTCTATTTTCGCTAAGTCACCGCCCCGAGATCTGTTTTCGCTGAGTCACGGTCCCG

Rev_2

GTGTCTGTTTTCGCTGAGTCACGGTCTAGAGATTTGTTTTCCTCAGAGTTCCAGCTGCTCCAG

Determined hHO-1 eRNA E2 5' end

GTTTAATCCCCTGGGGCAAAGTCCGGACTGTCCGGCTGGAGTCTGGAGTCGGGACATGCCTCA

TCID:T22F020836C6

GCCAGCACGTCCTCGGCCTCGTCTGGGGCCTGAATCCTAGGGAAGCCATAGCAGCTCCTCCAC

Fwd_3

CCTTCCTCTCACTCCTCCTCTAGCCTCTTGCTACTCCCCGCACCACTGTTTTAGGGAACCTCT
ATCTCCCGACGGCCTGCCACGGGCCAGGCGCTGTGCTGGGGGCTTCACACTTTAAATCGCTGT
TGAGCGGGGCGCGGGGGCGCTGCAACCTAAAGGTGGGAGCTACTCAA**ATGGAGGGGCATCTGT**
TAAAATGGCCGGCCTGTCATTTTCAAAAACTTCAAGGCCGGGCGCGGTGGCTCACGCCTGTAA
TCCCAGCACTTTGGGAGGCCGAGGCGGGCGGATCACGAGGTCAGGAGATCGAGATCATCTTGT

TCID:T22F0208379A

CTAACACGGTGAAACTTCATCTCTACTAAAAATAGAAAAAATTAGCCGGGCGTGGTGGCGGGC

5' RACE reverse primer (5' RACE E2 Reverse_2)

GCCTGTAATCCCAGCTACTCAGGAGGCTGAGGCAGGAGAATGGCATGAACCCGGGAGGCGGAG
CTTGCAGTGAGCCGAAATCGCGCCACTGCAGTCCGGCCTGGGCGAAAGAGCAAGACTCCGCCT
CAAAAAAAAAAAAAAAAAAAAACTTCAAAGGCTGAGGAACCCAAAGAGGCAGGACAAGTGAAT

Rev_3

GCAATGCAACCTCTTGGGCTGGAACCTGGACTGGTAAAACGGCTAAAGAGGAGGTTATTGGGG

Figure S4 DNA sequence and conservation of *hHO-1 eRNA E2s*.
5' RACE analysis was performed using total RNA from DEM-treated HeLa cells and the DNA sequences of obtained the 5' RACE clones were determined. (A) The 5' RACE reverse primer (pink arrow) is shown with the primer sets used in the RT-PCR analysis above the schematic representation of the *HO-1* E2 enhancer region. The determined hHO-1 eRNA E2 5' end is indicated by a black arrow. (B and C): Conservation tracks of the region around the E2 enhancer (B) and the region adjacent to the putative hHO-1 eRNA E2 5' end (C) were obtained from the UCSC Genome Browser. In (B), the core *HO-1* E2 enhancer region is underlined in pink and the determined hHO-1 eRNA E2 5' end is shown by a pink dot. (D) The DNA sequence adjacent to the human *HO-1* E2 enhancer region and the primers used in this study are shown. TCIDs represent the transcription start sites in the CAGE data. Green letters represent the DNA portions that encode the possible ORF.

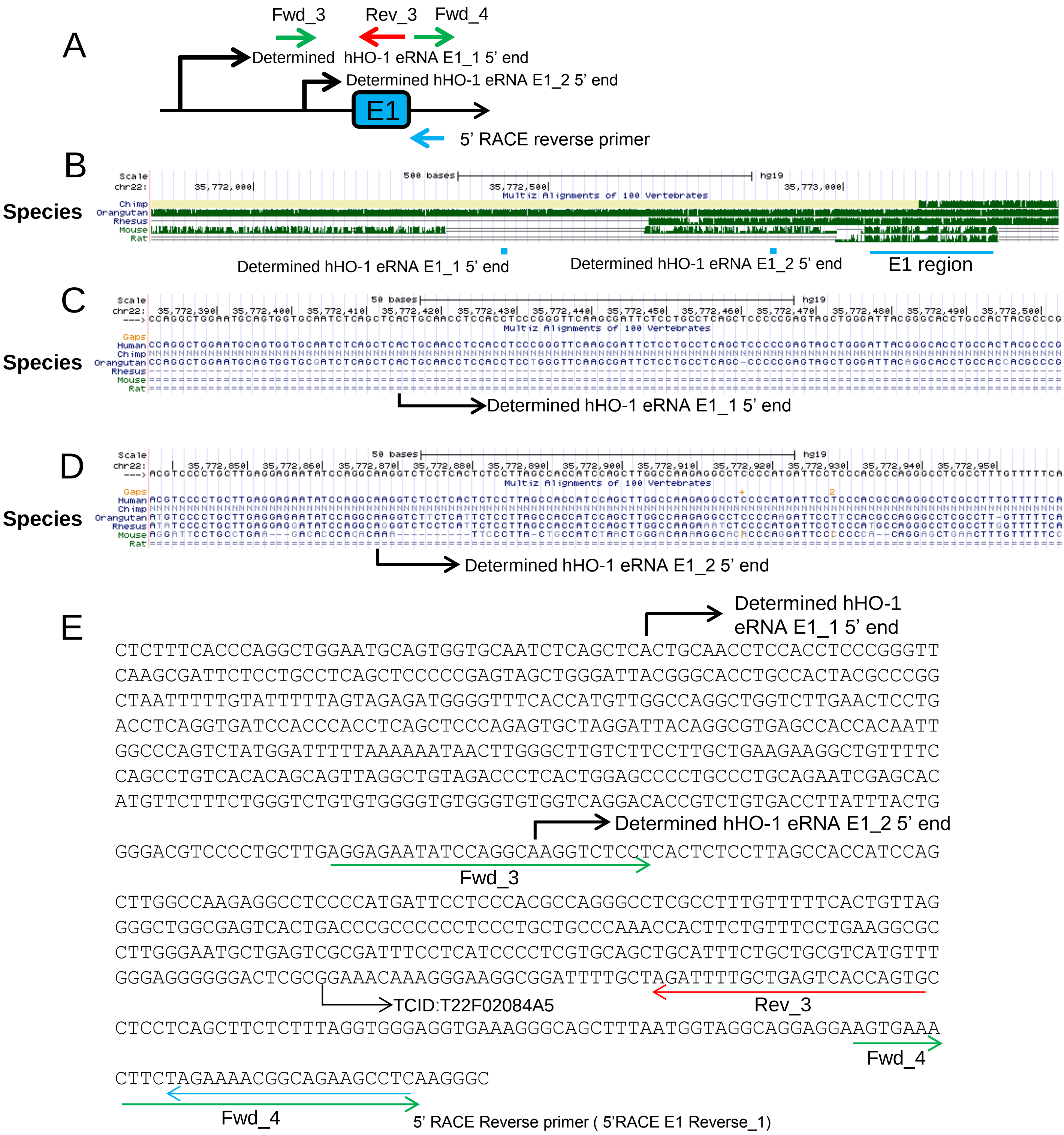

Figure S5 Sequences and conservation of *hHO-1 eRNA E1s*.
5' RACE was performed using total RNA from DEM-treated HeLa cells as a template and a 5' RACE reverse primer (cyan arrow). A schematic figure of the human *HO-1* E1 enhancer region, the primers used in 5' RACE (A) and its conservation in vertebrates (B, C and D) are shown. Conservation tracks in the region of the E1 enhancer (B) and adjacent to the putative *eRNA E1s* 5' ends (C and D) were obtained from the UCSC Genome Browser. The determined DNA sequence and the 5' ends are indicated by black arrows. (E) The detailed DNA sequence adjacent to the human *HO-1* E1 enhancer region and the primers used in this study are shown. TCID represents the transcription start site in the CAGE data.

Supplemental Figure S6

Maruyama et al.

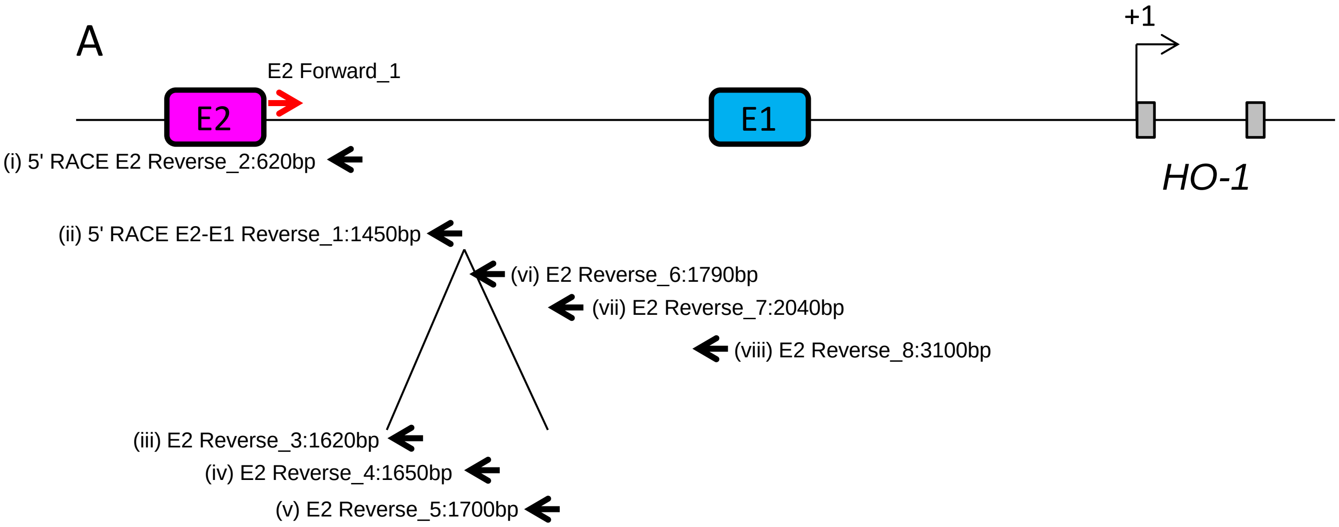

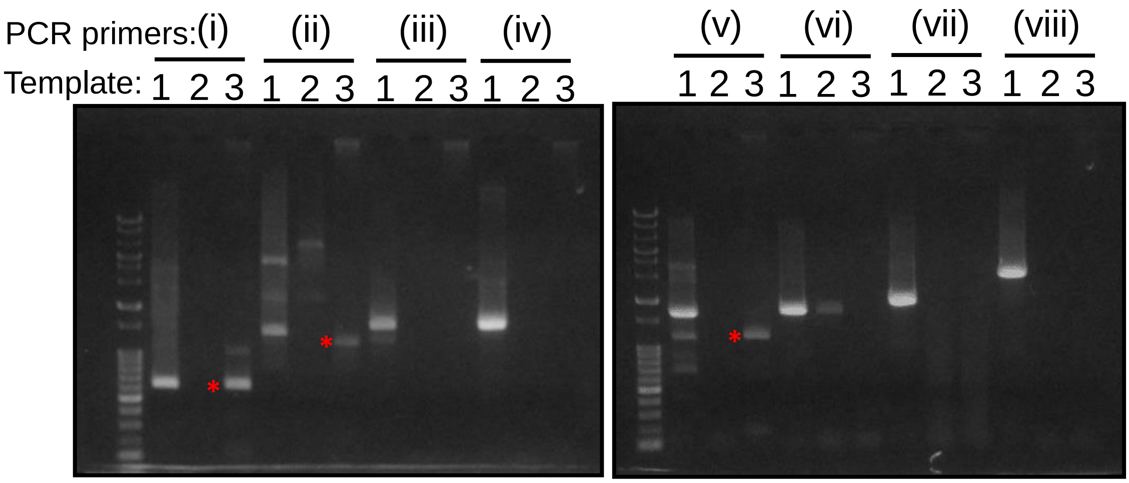

Figure S6 3' primer walking analysis of *hHO-1 eRNA E2-3*.
To estimate the full-length of *eRNA E2-3*, cDNAs synthesized using random hexamers and total RNA of DEM-treated HeLa cells were examined by 3' primer walking analysis. (A) The location of the primers used in 3' primer walking analysis are shown. (A) A downward E2 Forward_1 primer (red arrow) and a series of upward primers ((i) to (viii)) were used. The primer sequences are listed in Supplementary Table S1. The sizes of the PCR products are indicated to the right of the primer name. (B) Ethidium bromide staining of PCR products in 3' primer walking analysis. The PCR primers used for analysis are indicated in the figure. We performed RT-PCR using the following templates: 1: genomic DNA of HeLa cells; 2: RTase minus with random hexamer and DEM-treated HeLa total RNA; 3: RT reaction with random hexamers and DEM-treated HeLa total RNA. Amplified RT-PCR products (asterisks) were subcloned using a ZeroBlunt TOPO PCR cloning Kit and the DNA sequence was analyzed.
A PCR fragment of (v)- lane 3 contained *hHO-1 eRNA E2_L*. We also confirmed by DNA sequencing that the PCR fragment of (i)-lane 3 contained DNA sequence corresponding to *eRNA E2*-3, whereas the PCR fragment of (ii)-lane 3 was a non-specific PCR amplification.

Supplementary Figure S7

Maruyama et al.

Human *HO-1* genomic region

A

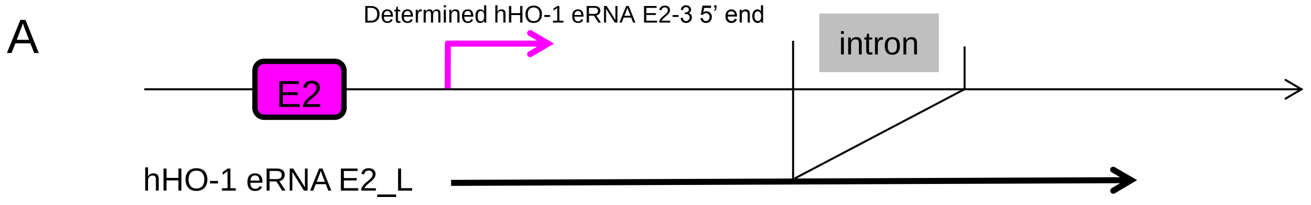

B

```
   1                                         GGGGCAAAGTCCGGACTGTCCGGCTGG 27
  28 AGTCTGGAGTCGGGACATGCCTCAGCCAGCACGTCCTCGGCCTCGTCTGGGGCCTGAATC 87
                                                        E2 Forward_1
  88 CTAGGGAAGCCATAGCAGCTCCTCCACCCTTCCTCTCACTCCTCCTCTAGCCTCTTGCTA 147
 148 CTCCCCGCACCACTGTTTTAGGGAACCTCTATCTCCCGACGGCCTGCCACGGGCCAGGCG 207
 208 CTGTGCTGGGGGCTTCACACTTTAAATCGCTGTTGAGCGGGGCGCGGGGGCGCTGCAACC 267
 268 TAAAGGTGGGAGCTACTCAAATGGAGGGGCATCTGTTAAAATGGCCGGCCTGTCATTTTC 327
 328 AAAAACTTCAAGGCCGGGCGCGGTGGCTCACGCCTGTAATCCCAGCACTTTGGGAGGCCG 387
 388 AGGCGGGCGGATCACGAGGTCAGGAGATCGAGATCATCTTGTCTAACACGGTGAAACTTC 447
 448 ATCTCTACTAAAAATAGAAAAAATTAGCCGGGCGTGGTGGCGGGCGCCTGTAATCCCAGC 507
 508 TACTCAGGAGGCTGAGGCAGGAGAATGGCATGAACCCGGGAGGCGGAGCTTGCAGTGAGC 567
 568 CGAAATCGCGCCACTGCAGTCCGGCCTGGGCGAAAGAGCAAGACTCCGCCTCAAAAAAAA 627
 628 AAAAAAAAAAAAACTTCAAAGGCTGAGGAACCCAAAGAGGCAGGACAAGTGAATGCAATG 687
                                         (i)5'RACE E2 Reverse_1
 688 CAACCTCTTGGGCTGGAACCTGGACTGGTAAAACGGCTAAAGAGGAGGTTATTGGGGCAA 747
 748 TAGGGGACATTTGAATATAGGCTTTATATTGAAGGAGTTCAGGATATGCCACCCAAAATG 807
 808 TGCCACTTTGGATTAAGGATCATTATTATTATTATTATTATTATTTGAGACAGGGTCTC 867
 868 TGTCACCCAAGCTGCAGTGCAGTGGCACAATCTCGGCTCACTGCAACCTCTGCCTCCTAG 927
 928 GTTCAAGCGATTCTCGTGCCTCAGCCTCCCAAGTAGCTGCGATTACAGGTGTGTGCCACC 987
 988 ACTCCCCATTCATTGATTAAGGATTATTTTGAGCTGGCCAGGCGTGGTGGCACATGCCTG 1047
1048 TAATCCCAGCACTTGGGGAGGCCACTGTGGGGGGATTGCTTGAGCTCAGGAGTTCCAGAC 1107
1108 CAGCTTGGGCAACGTGGTGAAACCTTGTCTCTACAAAAAATACAAAATATAAAAAATTTG 1167
1168 CCAGGCATGGTGGTGCATGTCTATAATCCCAGCTACTTGGGAGGCTGAGGCAGGAGGATT 1227
1228 GCTTGAGCCCAGGAGATTGAGGTTGCAGACCACTACACTCCAGCCTGGGAAACAGAGTGA 1287
1288 GACTCTGTCTCAAAAAAAAGGATTATTTTGGGATAAAGGCACTTTAAAAACAAACAAACA 1347
1348 AAATCAGGTGCAAAAAGGTTGCTGTGGGTTGGGCATGGTCGCTCAGGCCTGTCATCCCAG 1407
1408 CACTTTGGGAGGCAAAGGCAGGCAGACCACCTGAGGTCAGGAGTTCGAGACCAGCCTGAC 1467
1468 CAACTTGGAGAAACCTCATCTCTACTAAAAATACAAAATTATCCGGGCGTGGTGGCGCAT 1527
                                   (ii)5'RACE E2-E1 Reverse_1
1528 GCCTGTAATCCCAGCTACTAGGGAGGCTGAGGTAGGAGAATTGCGTGAACCTGGGAGGCA 1587
1588 GAGGTTGCAGTGAGCCTCTGACCTTCTTTTTTTCTTCCTGAAAGCAAGAGATGAAATTCC 1647
1648 ATATGGAAGACTTTTCTCTATACGAGGAGAAAATTAGCATTCCTTTCATCCATGGCAGGA 1707
                           (iii) E2 Reverse_3             (iv) E2 Reverse_4
1708 AGTTGAAGCCAAAATAAATCTGTACAAACAAAGCTTGTTAAAGCCAGCTCTTCCTAGTCT 1767
                                                   (v) E2 Reverse_5
1768 CT...                                                        1769
```

Figure S7 DNA sequence of *hHO-1 eRNA E2_L*.
We performed a 3' primer walking analysis as shown in Figure S6 and the DNA sequences of the plasmid clones were determined. (A) A schematic representation of the putative transcript (hHO-1 eRNA E2_L) is shown by bold black arrows. (B) The sequence obtained from the subclone of PCR using E2 Forward_1 and (v) E2 Reverse_5 is underlined against the *HO-1* E2 enhancer region sequences. Putative intron is indicated by hatching. We identified CAGC sequences at the both sides of the intron/exon junction of genomic DNA (shown in red). The hHO-1 eRNA E2_L contains only one of these. The DNA sequence that encode the putative ORF is indicated by green letters. The 5' end of *hHO-1 eRNA E2-3* is indicated by a magenta letter. The primers used in this experiment are shown by arrows below the sequence.

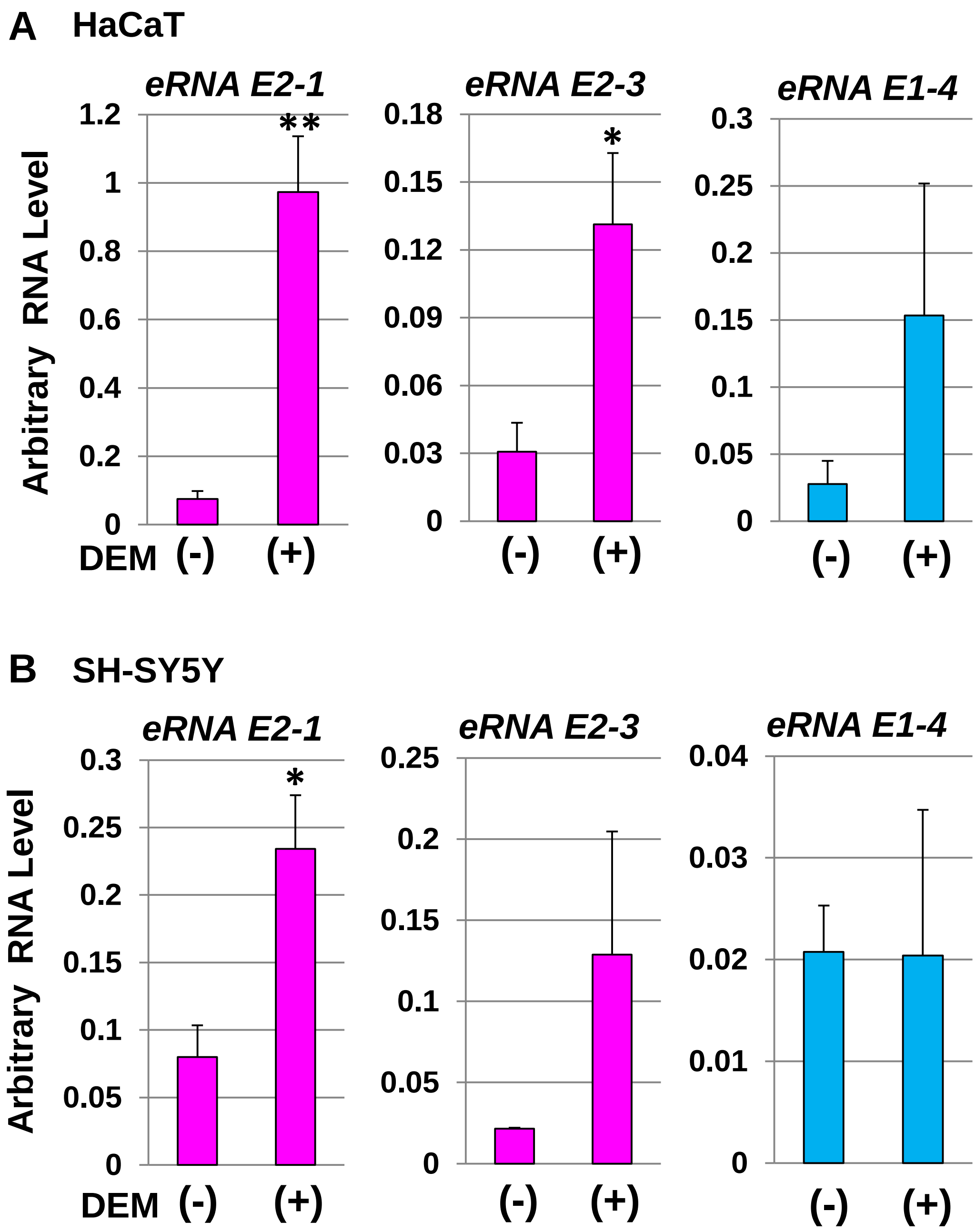

Figure S8 ***hHO-1 eRNAs* expression in human cultured cells.**
We analyzed the expression of *hHO-1 eRNAs* in HaCaT and SH-SY5Y cells. HaCaT (A) and SH-SY5Y (B) cells were either untreated (-) or treated with 100 μM DEM for 6 hours (+) and total RNA samples were isolated. The cDNAs were synthesized using random hexamers with total RNA as a template. The arbitrary RNA levels of *eRNA E2-1*, *eRNA E2-3* and *eRNA E1-4* were measured by real-time RT-PCR using specific primers and Universal Probe Library Probes. The value was normalized to the expression of the cyclophilin A gene, and the arbitrary RNA level was expressed as the mean ± SEM of three independent assays. *: *P*<0.05; **: *P*<0.01 (two-tailed unpaired Student's *t*-test).

Supplementary Figure S9

Maruyama et al.

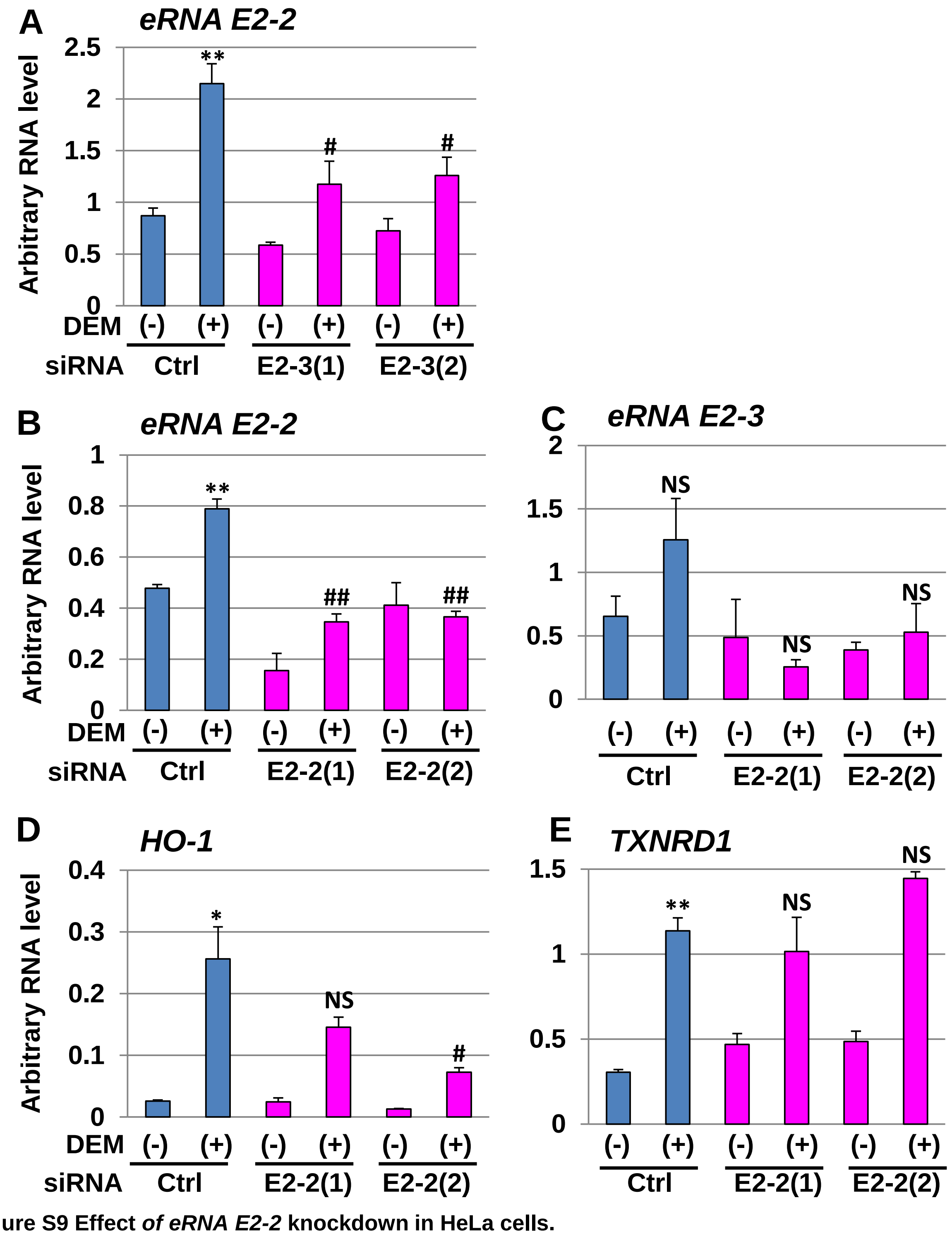

Figure S9 Effect *of eRNA E2-2* knockdown in HeLa cells.
(A)The effect of *eRNA E2-3* knockdown (KD) on *eRNA E2-2* expression. (B and C) The effect of *eRNA E2-2*-KD on *eRNA E2-2* (B) or *eRNA E2-3* (C) expression. HeLa cells were transfected with control siRNA (Ctrl) or two siRNAs against *eRNA E2-2* (E2-2(1) or E2-2(2)). Then the cells were either untreated (-) or treated with100 μM DEM for 6 hours (+). The RNA level was analyzed by real-time RT-PCR using specific primers and Universal Probe Library Probes. The effect of *eRNA E2-2*-KD on the expression of *HO-1* (D) and *TXNRD1* (E). The expression of *HO-1* and *TXNRD1* was analyzed by real-time RT-PCR. Each value was normalized to cyclophilin A gene expression and the arbitrary RNA level was expressed as the mean ± SEM of three independent assays. *: $P<0.05$; **: $P<0.01$ (two-tailed unpaired Student's *t*-test) compared to control siRNA without DEM (Ctrl (-)). #: $P<0.05$, ##: $P<0.01$; NS: no significance compared to the value of control siRNA with 100 μM DEM for 6 hours (Ctrl (+)) (one-way ANOVA followed by a Dunnett's *post-hoc* test for multiple parameter comparisons).

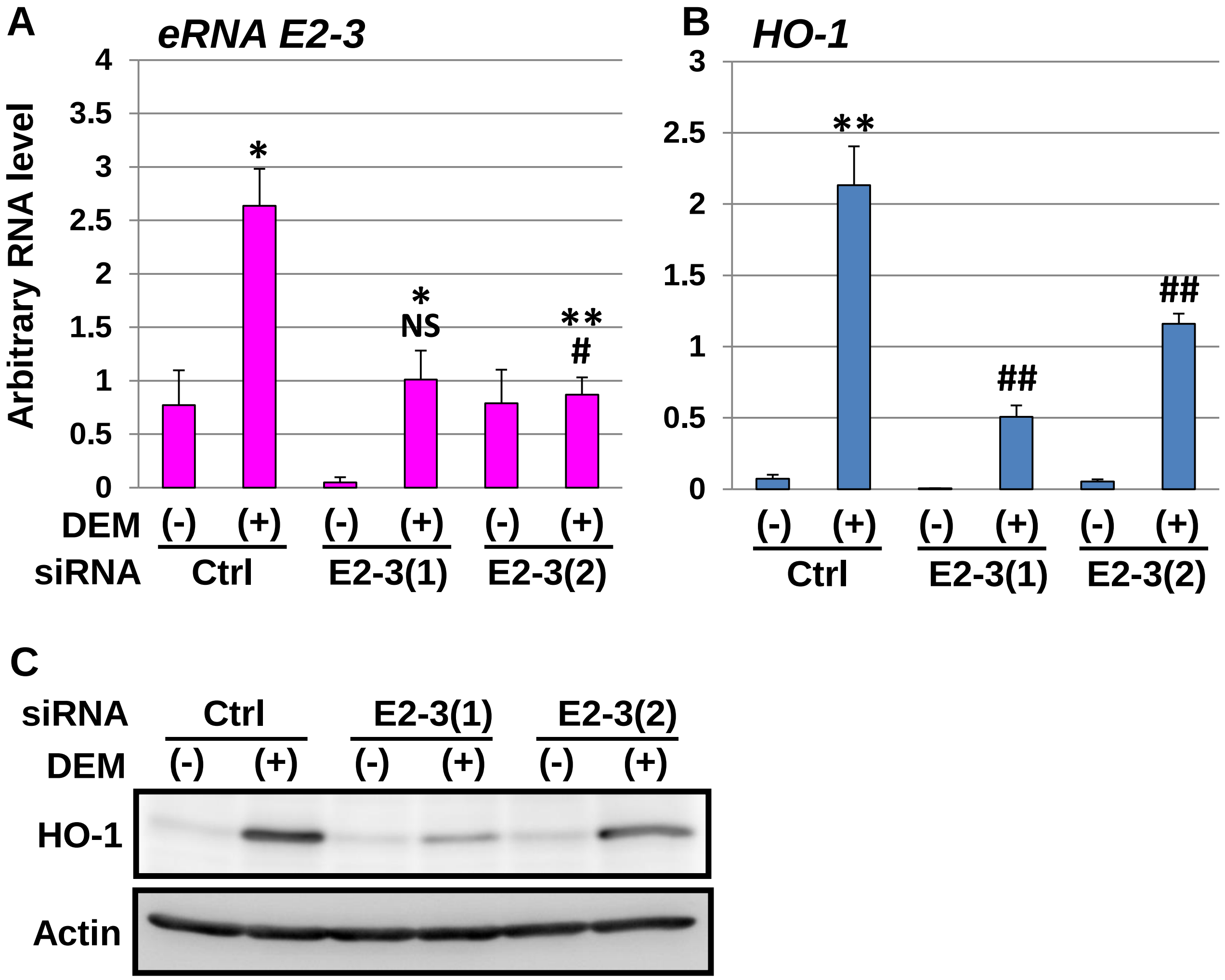

Figure S10 Effect of *eRNA E2-3* knockdown on *HO-1* expression in HaCaT cells.
(A) The effect of *eRNA E2-3* knockdown (KD) on *eRNA E2-3* expression in HaCaT cells. HaCaT cells were transfected with control siRNA (Ctrl) or two siRNAs against *eRNA E2-3* (E2-3(1) or E2-3(2)). The cells were untreated (-) or treated with100 μM DEM for 6 hours (+). The *eRNA E2-3* level was analyzed by real-time RT-PCR using specific primers and Universal Probe Library Probe #17. (B) The effect of *eRNA E2-3*-KD on the expression of *HO-1*. The expression of *HO-1* was analyzed by real-time RT-PCR. Each value was normalized to cyclophilin A gene expression, and the arbitrary RNA level was expressed as the mean ± SEM of four independent assays. *: P<0.05; **: P<0.01 (two-tailed unpaired Student's t-test) compared to control siRNA without DEM (Ctrl (-)). #: P<0.05; ##: P<0.01; NS: not significant compared to the value of control siRNA with 100 μM DEM for 6 hours (Ctrl (+)) (one-way ANOVA followed by a Dunnett's *post-hoc* test for multiple parameter comparisons). (C) The effect of *eRNA E2-3*-KD on HO-1 protein expression in HaCaT cells. Whole-cell lysates were separated by SDS-PAGE, and HO-1 protein expression was analyzed by immunoblotting using a HO-1-specific antibody (Abcam, ab68477). Actin was used as a loading control (Sigma-Aldrich, A1978).
